# Relevance of Peroxisome Proliferator Activated Receptors in Multitarget Paradigm Associated with the Endocannabinoid System

**DOI:** 10.3390/ijms22031001

**Published:** 2021-01-20

**Authors:** Ana Lago-Fernandez, Sara Zarzo-Arias, Nadine Jagerovic, Paula Morales

**Affiliations:** Medicinal Chemistry Institute, Spanish Research Council, Juan de la Cierva 3, 28006 Madrid, Spain; ana@iqm.csic.es (A.L.-F.); s.zarzoarias@gmail.com (S.Z.-A.)

**Keywords:** PPAR, cannabinoids, CB_1_R, CB_2_R, FAAH, multitarget, endocannabinoid system

## Abstract

Cannabinoids have shown to exert their therapeutic actions through a variety of targets. These include not only the canonical cannabinoid receptors CB_1_R and CB_2_R but also related orphan G protein-coupled receptors (GPCRs), ligand-gated ion channels, transient receptor potential (TRP) channels, metabolic enzymes, and nuclear receptors. In this review, we aim to summarize reported compounds exhibiting their therapeutic effects upon the modulation of CB_1_R and/or CB_2_R and the nuclear peroxisome proliferator-activated receptors (PPARs). Concomitant actions at CBRs and PPARα or PPARγ subtypes have shown to mediate antiobesity, analgesic, antitumoral, or neuroprotective properties of a variety of phytogenic, endogenous, and synthetic cannabinoids. The relevance of this multitargeting mechanism of action has been analyzed in the context of diverse pathologies. Synergistic effects triggered by combinatorial treatment with ligands that modulate the aforementioned targets have also been considered. This literature overview provides structural and pharmacological insights for the further development of dual cannabinoids for specific disorders.

## 1. Introduction

The concept of designed multiple-targeting ligands seems to appear with modern drug discovery approaches and especially in 2004 when Morphy et al. [1] published “From magic bullets to designed multiple ligands”. Over the past years, this emerging polypharmacological-based therapeutic approach has been explored for multifactorial diseases, for example Alzheimer’s disease, multiple sclerosis, diabetes, coronary heart disease, cancer, and rheumatoid arthritis [2,3,4]. Synergistic effects while reducing side effects are the pursued key goals compared to combination therapies. Nowadays, better understanding of protein/ligand complexes allows rationalizing the design of new multitarget ligands [3,5]. In this context, the endocannabinoid system (ECS) by itself represents a goldmine in multitarget therapeutic strategies.

**Figure 1:**
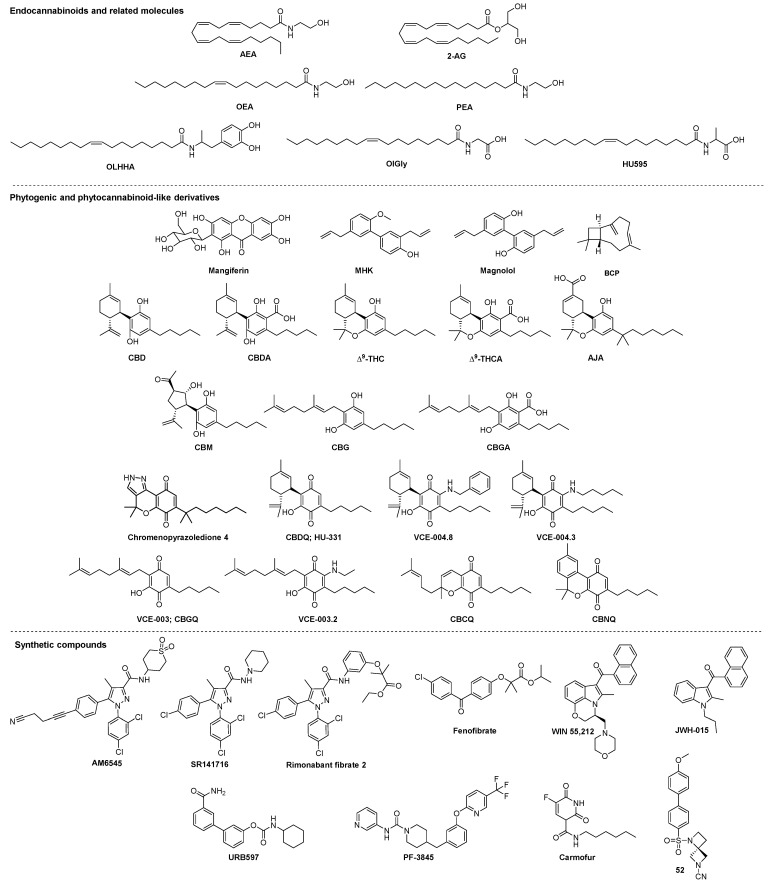
Structures of molecules modulating cannabinoid receptors (CBRs) and peroxisome proliferator-activated receptors (PPARs).

**Figure 2:**
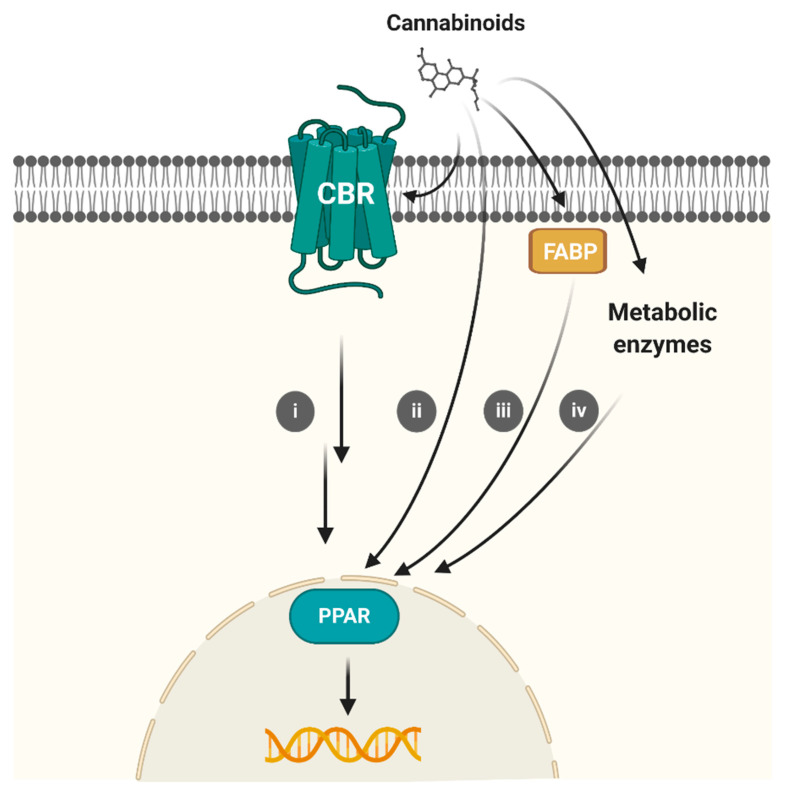
Representation of the mechanisms through which cannabinoids may interact with CBRs and PPARs. Cannabinoids diffuse through the cell membrane to reach CBRs. Different options have been postulated for cannabinoids–PPARs interactions: (**i**) CBR intracellular signaling pathways; (**ii**) Direct binding; (**iii**) Fatty acid-binding proteins (FABPs)-mediated transportation; (**iv**) Conversion to PPAR active metabolites. Created with BioRender.com.

Three decades ago, the first elements of the endocannabinoid signaling system were discovered thanks to Δ^9^-tetrahydrocannabinol (THC: Figure 1), one of the two major components of the plant *Cannabis sativa*, the other one being cannabidiol (CBD; Figure 1) [6]. The identification of the principal biological target of THC, the CB_1_ cannabinoid receptor (CB_1_R) [7], led to the discovery of two endocannabinoids, *N*-arachidonoyl-ethanolamine (AEA; anandamide, Figure 1) and 2-arachinoylglycerol (2-AG, Figure 1) a few years later [8,9,10]. Then, a second cannabinoid receptor, CB_2_R, was identified [11]. Thus, in the early part of the last decade, ECS was considered to comprise two cannabinoid receptors, CB_1_R and CB_2_R, the endocannabinoids AEA and 2-AG, and enzymes responsible for their degradation, the fatty acid amide hydrolase (FAAH) and the monoacylglycerol lipase (MAGL) (see [12] for a review). The ECS has been shown to regulate a variety of physiological and pathological activities, such as appetite, pain, memory, and inflammation [12]. Much progress in the understanding of the ECS has been made in the past few years. This includes a better knowledge of the signaling mechanisms and structural features of cannabinoid receptors (CBRs) [13], the mediator network involved in endocannabinoid metabolism, and the discovery of other G protein-coupled receptors (GPCRs), ion channels, and nuclear receptors as targets for cannabinoids [14].

CB_1_R and CB_2_R are very attractive and validated therapeutic targets of the ECS. CB_1_R is the most abundant GPCR in the brain with expression in the cortex, basal nuclei, hippocampus, and cerebellum. CB_1_R is also expressed in peripheral organs such as liver, kidney, heart, adipose tissue, muscle, lung, pancreas, and immune cells such as monocytes and macrophages. CB_1_R is considered a promising target for the treatment of different pathologies, including neurodegenerative diseases, metabolic syndromes, and neuropathic pain associated with multiple sclerosis and spinal cord injuries. CB_2_R is predominantly expressed in immune cells, included lymphocytes, natural killer cells, macrophages, and neutrophils but are also present in the brain, in brain microglia for instance. CB_2_R represents an attractive target for the treatment of inflammatory processes. Moreover, the modulation of CB_2_R does not involve the psychoactive adverse effects produced by the activation of CB_1_R in the brain. Much progress has been made in the structural elucidation of CBRs and their signaling complexes with G proteins. In the last 4 years, high-resolution crystal structures of human CB_1_R and CB_2_R in complex with antagonists [15,16,17], with agonists [18,19], and with an allosteric modulator [20] have been resolved. Cryo electron microscopy (cryo-EM) techniques allowed resolving the structure of CB_2_R and CB_1_R in complex with G_i_ proteins [19,21,22]. Li and colleagues [23] have recently published an overview on the structural features of CBRs in different functional states and the diverse ligand binding modes that could be largely explored in the context of multiple-ligand strategies.

Modulating the level of the two signaling lipids, AEA and 2-AG, is also a very attractive therapeutic strategy. However, as mentioned by Di Marzo [13], manipulating endocannabinoid levels without affecting other biochemically related mediators is often difficult. Effectively, the endocannabinoids belong to a complex network that includes the main enzymes involved in their biosynthesis and degradation, FAAH, MAGL, *N*-acyl phosphatidylethanolamine-specific phospholipase D (NAPE-PLD), diacylglycerol lipases α and β (DGLα, DGLβ), with other contributions such as α/β hydrolase domain containing 6/12 (ABHD6/12), endocannabinoid membrane transporter (EMT), or fatty acid binding proteins (FABP5) (see [13] for a comprehensive review of the endocannabinoidome mediators). Dual FAAH–MAGL inhibitors have already been developed at the preclinical stage such as JZL195 that shows synergic effects in vivo by the simultaneous blockade of these enzymes compared to selective inhibition [24].

Other GPCRs, ion channels, and nuclear receptors have been related to the ECS mainly due to the fact that some cannabinoids directly modulate their activity or exert physio or pathological functions mediated by these receptors and channels [25]. Among these GPCRs, the orphans GPR55 and GPR18 are particularly relevant, and they have been postulated to be putative cannabinoid receptors [26]. This is also the case for the transient receptor potential (TRP) channels TRPV1-TRPV4, TRPA1, and TRPM8 that have been termed the ionotropic cannabinoid receptors [27]. The ionotropic glycine receptors (GlyRs) are also involved in the ECS [28]. For instance, they have been shown to contribute to cannabinoid-induced analgesia [29]. Evidence supports that cross-talk between CBRs and some GPCRs may rely on the formation of heteromeric complexes including adenosine A2A receptor-CB_1_R [30,31], dopamine receptor 2-CB_1_R [32], δ opioid receptor-CB_1_R [33], and serotonine 5-HT_1A_-CB_2_R [34]. In this review, we will emphasize the role of the peroxisome proliferator-activated receptors (PPARs) in relation with the ECS and how these nuclear hormone receptors offer diverse multitarget opportunities within ECS.

PPARs are transcriptional effectors involved in regulating biological processes such as lipid metabolism [35], energy balance, adipogenesis, inflammation [36], cell growth, differentiation, and apoptosis [35]. Thus far, three subtypes have been identified: PPARα, PPARβ/δ, and PPARγ [37]. They are ligand-dependent transcription factors that regulate target gene expression by binding to specific peroxisome proliferator response elements (PPREs). PPARs bind to its corresponding PPRE as heterodimers with a retinoid X receptor (RXR). Thus, upon binding an agonist, the alteration of the PPAR conformation produces the recruitment of transcriptional coactivators, leading to an increase in gene transcription [37]. The activities of PPARs are mainly associated with fatty acid oxidation and metabolism. PPARα and PPARβ/δ are highly expressed in liver (PPARα), brown adipose tissue (PPARα), intestine (PPARβ/δ) heart, skeletal muscle, kidney, and skin. The two major isoforms of PPARγ, PPARγ1 and PPARγ2, are differently expressed being PPARγ1 expressed in numerous cells included immune and brain cells, whereas PPARγ2 is especially present in white and brown adipose tissues. Several fatty acid derivatives bind and/or activate PPARs. Fibrates and thiazolidinediones (TZDs) are two conventional classes of PPAR ligands (for recent developments of synthetic PPAR ligands, see [38]). Troglitazone, rosiglitazone, and pioglitazone are PPARγ agonists used for treatment of type 2 diabetes mellitus (T2DM). However, the first two have been withdrawn from the market by most countries due to hepatotoxicity and cardiovascular side effects respectively, pioglitazone not being exempt of serious side effects. Several PPARα agonists, including ciprofibrate, fenofibrate, and bezafibrate, are approved drugs in some countries with a clear risk of hepatotoxicity.

So far, no synthetic cannabinoids apart from synthetic THC are in the market due to adverse outcomes, mainly psychotropic effects (CB_1_R agonists and antagonists), lack of efficacy (CB_2_R agonists), or possible immunosuppression (CB_2_R agonists). Thus, emerging strategies are being explored in academic and pharmaceutical laboratories [14]. One of these strategies focuses on ECS-PPARs for developing multiple target therapeutic agents. Comprehensive reviews from O’Sullivan et al. [31,39,40] describe the activation of PPARs by some cannabinoids including endocannabinoids, phytocannabinoids, and synthetic cannabinoids, and cannabinoids’ therapeutic effects are mediated by PPARs. The mechanisms of action involved in the cannabinoids/PPARs interactions are not yet being resolved, even though there has been increasing evidence supporting the ability of PPARs activation to mediate some of the therapeutic effects of cannabinoids over the past 15 years. 

CBRs and PPARs have shown to exhibit biological relevance in common pathophysiological contexts. For instance, both CB_1_R and PPARα have shown a therapeutic role in the regulation of lipid metabolism. Moreover, because of their immunomodulatory profile, CB_2_R and PPARγ have been widely studied for the management of diverse inflammatory diseases. These shared properties evidence the potential of multitargeting strategies in the context of specific diseases.

In this review, we will explore the connection between PPARs and elements of the ECS through the action of different cannabinoids reported so far to be engaged in this relationship. 

## 2. CB_1_R–PPAR Modulation

Certain therapeutic outcomes triggered by cannabinoids have shown to be mediated through the modulation of CB_1_R along with the PPAR subtypes PPARα or PPARγ. Dual targeting CB_1_R/PPAR as well as co-administration have been reported as beneficial pharmacological strategies in the course of numerous pathologies including obesity, arthritis, cancer, epilepsy, and alcohol use disorder [31,41,42]. 

### 2.1. CB_1_R–PPARα

Concomitant actions at CB_1_R and PPARα receptors have shown to be involved in the therapeutic effects exhibited by cannabinoids in metabolic syndrome. CB_1_R blockage is widely known for the reduction of body weight gain in obese subjects [43,44,45]. Moreover, PPARα activation has shown to play a therapeutic role in the regulation of lipid metabolism and obesity [46,47]. Taking advantage of these modulatory profiles, diverse studies have pursued CB_1_R/PPARα dual targeting to tackle obesity. For instance, fatty acid amide derivatives conjugated with amphetamines have been claimed as CB_1_R/PPARα dual ligands with antiobesity properties [48,49]. The most potent compound of this series, the oleic acid–dihydroxyamphetamine (OLHHA) (Figure 1), a weak CB_1_R antagonist and moderate PPARα agonist, proved to induce satiety and control food intake, reduce body fat, and regulate fat metabolism in rats [48,49]. As demonstrated by these authors, molecular mechanisms mediating OLHHA antiobesity effects involve CB_1_R and PPARα, while FAAH and TRPV1 receptors do not contribute to food intake modulation. 

Further investigations using OLHHA revealed its ability to improve non-alcoholic fatty liver disease (NAFLD) in an genetic animal model of obesity [50]. In this study, the immunohistochemical and histological analysis of liver and plasma samples of lean and obese Zucker rats upon OLHHA chronic treatment confirmed its anti-steatotic and hepatoprotective profile. A significant decrease in hepatic lipid accumulation, reduction of plasma levels of triglycerides, and cholesterol along with anti-apoptotic activity was also observed in obese rats. These hepatoprotective properties were related to an increase of CB_1_R expression and a downregulation of lipogenesis-related enzymes, while changes in PPARα mRNA expression between treated and vehicle rats were not significant. Even though these studies evidence the therapeutic potential of OLHHA, additional research is needed to fully determine its mechanism of action in obesity-associated fatty liver.

It is worth noting that the CB_1_R/PPARα dual profile of OLHHA was also proved to be effective at reducing alcohol intake [51]. In animal models of alcohol consumption, treatment with OLHHA was able to significantly reduce alcohol self-administration and voluntary alcohol consumption without triggering tolerance or toxicity. Therefore, this compound is a promising lead not only for the management of eating disorders and associated pathologies but also to treat alcohol use. 

In an effort to obtain dual ligands targeting CB_1_R and PPARα, the diarylpyrazole core of the CB_1_R antagonist/inverse agonist SR141716A (Rimonabant, Figure 1) was fused to the phenoxypropanoate pharmacophore of the fibrates (fenofibrate, PPARα agonist, Figure 1), obtaining the so-called rimonabant fibrates [52]. The most potent compound of this series, **2** (Figure 1), exhibited nanomolar activity as a PPARα agonist in luciferase reporter gene assays and a CB_1_R antagonist in mouse vas deferens contractile response assays. Even though its therapeutic potential has not been proved yet, this dual ligand may impair metabolism through different molecular mechanisms.

Another therapeutic strategy involving both targets was developed upon the peripheral blockade of CB_1_R in a diet-induced obese (DIO) mouse model [53]. The peripherally restricted antagonist AM6545 was able to reduce hepatic steatosis and improved liver injury through PPARα as shown by its lack of activity in PPARα knockout DIO mice. However, AM6545 failed to directly bind or modulate PPARα. Thus, the antisteatotic effects of AM6545 are not the result of direct dual targeting but rather due to the ability of CB_1_R to regulate hepatic PPARα. The authors suggested that antagonizing CB_1_R by AM6545 may increase the levels of hepatic endocannabinoid-like compounds, oleoylethanolamide (OEA, Figure 1) and palmitoylethanolamide (PEA, Figure 1), which may then directly activate PPARα [53].

Evidence from animal models shows that the combinatorial treatment of OEA and SR141716A is a successful approach for the control of obesity [54]. OEA is a shorter monosaturated analogue of AEA that has shown to activate PPARα while being devoid of CBR activity. This endogenous PPARα agonist had already been reported to reduce body weight and regulate satiety via this nuclear receptor [55,56]. The combinational therapy of the aforementioned CB_1_R antagonist with OEA in obese Zucker rats resulted in an improved reduction on feeding, body weight gain, and cholesterol levels along with an inhibition of enzymes involved in lipid biosynthesis, evidencing the synergistic effects of both drugs [54]. These results support combining both mechanisms of action to provide a more efficient treatment for the management of obesity.

In addition to obesity, other pathologies have shown to be impacted by mechanisms that involve CB_1_R and PPARα modulation. The activation of these targets has been respectively studied for their neuroprotective effects [41,57,58,59,60]. An example of dual CB_1_R and PPARα activation in this context is the antiepileptic profile of the endocannabinoid PEA [61]. PEA, a non-saturated analogue of OEA, exhibited anti-absence effects in a rat model of absence epilepsy (WAG/Rij rats), which were reversed by SR141716A and by the PPARα antagonist, GW6471. This endogenous compound is a PPARα agonist but lacks affinity toward the cannabinoid receptors CB_1_R and CB_2_R [31,62]. Therefore, these authors postulate that its CB_1_R-mediated antiepileptic effects are due to an enhancement of AEA activity by an *entourage effect* [61], which was also observed in previous PEA studies in other biological systems [63,64]. 

Likewise, the antinociceptive properties of certain cannabinoids have shown to be mediated through CB_1_R and PPARα. For instance, the endocannabinoid PEA exhibits analgesic effects via PPARα direct and CB_1_R indirect activation in an osteoarthritic chronic pain rat model [65]. Its behavioral effects were antagonized by SR141716A and GW6471; however, the implication of other targets, including the TRPV1 channel and the orphan GPCR GPR55, cannot be ruled out. In the same study, behavioral tests demonstrated that the antinociceptive properties of the synthetic cannabinoid agonists HU210 and WIN55,212-2 (Figure 1) are not due to a dual CB_1_R/PPARα mechanism but mainly mediated by the cannabinoid receptor. 

Moreover, the co-activation of both targets using the endocannabinoid AEA and the PPARα agonist GW7647 also demonstrated effective pain reduction [66]. Their synergistic effects significantly decreased pain behavior in a mouse model of acute chemical-induced pain.

### 2.2. CB_1_R-PPARγ

Pharmacological cannabinoid effects can also be mediated through CB_1_R/PPARγ dual mechanisms. Both receptors have shown to be involved in pathological processes including pain, tumor growth, or obesity [67]. PPARγ and CB_1_R have been associated to diverse types of cancer. Extensive research has proved the therapeutic utility of CB_1_R activation in the progress of a wide variety of tumors [68,69]. Moreover, the proapoptotic and antiproliferative properties of diverse cannabinoids have shown to be at least partially mediated by PPARγ activation [70]. Therefore, it is not surprising that the anticancer actions of specific cannabinoids are mediated through a dual mechanism. This antitumor CB_1_R/PPARγ profile can be exemplified by chromenopyrazoledione **4** (Figure 1), which is a cannabinoid quinone that exerts antiproliferative effects in hormone-sensitive prostate cancer in vitro and in a murine xenograft model [71]. Experiments in the androgen-sensitive LNCaP cell line demonstrated that this compound induces cancer cell death through a mechanism that involves PPARγ and CB_1_R activation as well as oxidative stress. Moderate CB_1_R binding affinity was reported, but direct PPARγ activation remains to be examined. Even though its ability to inhibit tumor growth was confirmed in prostate cancer xenograft mice, the suggested dual CB_1_R/PPAR mechanism needs to be confirmed in vivo.

The non-intoxicating phytocannabinoid CBD has also been reported to exert antitumor actions through CB_1_R and PPARγ in specific types of cancer [70,72,73]. Studies in colorectal carcinoma cell lines showed that CBD was able to significantly reduce cell proliferation via CB_1_R, PPARγ, and TRPV1 activation. Its ability to protect DNA from oxidative damage and enhance endocannabinoid levels were also observed upon CBD treatment [73]. CBD had been previously shown to bind and activate PPARγ [74]; nevertheless, weak CB_1_R activity was reported for this phytocannabinoid [75]. In light of that, the authors attribute CB_1_R-mediated antiproliferative effects in colon cancer cells to indirect activation due to endocannabinoids enhancement [73]. It is important to note that CBD antitumor properties in other types of cancer have not been related to dual CB_1_R and PPARγ [72]. For instance, in lung cancer cell lines, CBD mediates proapoptotic effects via PPARγ and COX-2 but not through CB_1_R, CB_2_R, or TRPV1, as demonstrated upon treatment with their corresponding antagonists [76]. 

Although strategies targeting CB_1_R/PPARα have been explored in further depth for the treatment of obesity, the concomitant modulation of CB_1_R and PPARγ may also be useful for the control of metabolic syndrome and related disorders. A recent study demonstrated that these two targets are involved in the antiobesity and antiadipogenic effects of leaves extracts from *Mangifera indica* (EMI) [77]. Treatment with EMI led to a reduction of food intake and adipose tissue in a rat model of cafeteria diet-induced obesity. These effects were accompanied by increased PPARγ and decreased CB_1_R mRNA expression. Curiously, the major bioactive component of EMI, the xanthone glycoside mangiferin (Figure 1), produced proadipogenic effects in the same rat model. The presence of other phenolic compounds identified in the extracts may enable the beneficial effects produced by EMI when compared to isolated mangiferin [77]. Studies to explore the direct modulation of CB_1_R and PPARγ with the extracts and with the isolated compounds remain to be done.

The role of CB_1_R and PPARγ has also been extensively demonstrated in inflammatory processes [78,79,80]. Indeed, Δ^9-^tetrahydrocannabinolic acid (THCA), a phytogenic precursor of THC, exerts anti-arthritis activity through CB_1_R and PPARγ pathways [81]. In a murine model of collagen-induced arthritis, THCA was able to significantly decrease inflammatory biomarkers, synovial hyperplasia, and cartilage damage. These effects were abolished upon treatment with either SR141716 or T0070907 (PPARγ antagonist). THCA direct activation of both CB_1_R and PPARγ was confirmed by competitive binding assays and functional studies [81,82]. At CB_1_R, it was shown to act as an orthosteric agonist or as a positive allosteric modulator in the presence of the synthetic agonist CP-55,940 [81]. It also behaves as a CB_2_R inverse agonist; however, this effect was not involved in Δ^9^-THCA anti-arthritis properties.

Collectively, these data demonstrate the therapeutic potential of CB_1_R–PPARα and CB_1_R–PPARγ pharmacological strategies in a wide range of pathologies. Approaches targeting these ECS receptors can effectively trigger synergistic actions that may offer better hopes in the course of diverse diseases. However, so far, clinical trials remain to be performed in order to confirm the results observed in animal models. A summary of CB_1_R-PPAR compounds associated by target and disease is displayed in Table 1.

## 3. CB_2_R–PPAR Modulation

Dual targeting CB_2_R/PPAR has also been explored as a promising therapeutic approach in the course of diverse diseases.

### 3.1. CB_2_R–PPARγ

Since CB_2_R and PPARγ share immunomodulatory properties, multitarget strategies for these receptors are primarily focused on pathologies with a pro-inflammatory component. In fact, several natural and synthetic dual ligands have shown promising results as therapeutic options in preclinical and clinical studies for arthritis, fibrotic diseases, multiple sclerosis, neurodegenerative disorders, substance abuse and mood disorders, cancer, and metabolic dysfunction (see Table 1). On the one hand, cannabinoids targeting CB_2_R show beneficial effects on rheumatoid arthritis by inhibiting inflammation and osteoclastogenesis in preclinical assays [126,127,128]. On the other hand, PPARγ is expressed in synoviocytes, where it regulates the expression of inflammatory mediators and osteoclast differentiation [129,130]. Ajulemic acid (AJA) (Figure 1) is a synthetic derivative of Δ^9^-THC-11-oic acid with beneficial results in rheumatoid arthritis, as shown in experimental in vitro and animal models. AJA reduces inflammation through both CB_2_R and PPARγ activation [83,131,132], leading to a significantly reduction in pro-inflammatory mediators [131,132,133,134], edema [133,135,136], synovitis [133], bone and cartilage damage [133,137], and osteoclastogenesis [138]. Similar effects were observed with β-caryophyllene (BCP, also trans-caryophyllene) (Figure 1), which is a selective CB_2_R phytochemical compound that also binds to PPARα and upregulates PPARγ in a CB_2_R-dependent manner [84,139,140]. BCP prevented inflammation and cartilage damage in an experimental animal model of arthritis by reducing pro-inflammatory cytokines and recovering PPARγ expression [84]. These effects were abrogated by AM630, a CB_2_R antagonist, suggesting CB_2_R-involvement. 

A combined multitarget approach can be a valuable strategy in other chronic autoimmune diseases, such as systemic sclerosis or scleroderma. CB_2_R agonists have shown anti-fibrotic and anti-inflammatory effects in animal models of fibrosis [141,142,143], whereas PPARγ agonists can block the pro-fibrotic transforming growth factor beta (TGF-β)/SMAD (small mothers against decapentaplegic) signaling pathway at the nuclear level [144]. Accordingly, AJA and the cannabidiol quinones VCE-004.3 and VCE-004.8 (also known as EHP-101 Figure 1), reduced the fibrotic and inflammatory response in animal models of systemic sclerosis by interfering with SMAD transcriptional activity and decreasing TGF-β-induced collagen synthesis and release [85,86,87,145]. Treatment with these dual compounds also modified the transcriptome signature, reducing gene expression of pro-inflammatory and pro-fibrotic signals [86,87,88,146]. AM630 was able to attenuate the effects of both quinones, while the PPARγ antagonist T0070907 only prevented VCE-004.8 effects but not the ones of VCE-004.3 [86,87]. However, that does not necessarily exclude PPARγ involvement, since both quinones and AJA bind at different site of the ligand-binding domain (LBD) than other canonical PPARγ ligands, including T0070907 [86,100]. These favorable effects have advanced these CB_2_R-PPARγ ligands into clinical trials with promising results regarding systemic sclerosis [146,147]. 

VCE-004.8 and BCP have shown very promising results over multiple sclerosis, which is an autoimmune demyelinating disease characterized by severe neuroinflammation [89,90,148,149,150]. Both ligands were able to significantly improve the clinical features in experimental animal models by reducing the exacerbated inflammation in the central nervous system (CNS) [89,90,148,149] and, in the case of BCP, by also decreasing oxidative stress. While the effects of VCE-004.8 over multiple sclerosis pathogenesis are CB_2_R- and PPARγ-independent [90], BCP beneficial effects were reported to depend on CB_2_R activation at lower concentrations and PPARγ activation at higher concentrations [150]. 

Multiple reports showed that BCP and the natural product 4′-*O*-methylhonokiol (MHK) (Figure 1) prevented neuronal cell death with subsequent cognitive improvement in several Alzheimer’s disease animal models [91,92,151,152,153,154,155,156]. These natural products exerted their therapeutic effects by decreasing oxidative stress and inflammation [91,92,153,155,156,157]. Moreover, oral treatment with MHK increased amyloid β clearance by modulating the expression of β-secretase, the amyloid precursor protein), and several amyloid β-degrading peptidases [91,152,153,154]. Although the molecular target through which MHK exerts these effects has not yet been determined, this neolignan shows affinity for cannabinoid receptors with a 50-fold selectivity over CB_2_R [158] while also binding to the LBD of both PPARα and PPARγ [159]. On the other hand, the therapeutic effects of BCP over Alzheimer’s disease rely mainly on CB_2_R activation, and to a lesser extent, on PPARγ activation [92,156]. In fact, BCP has shown neuroprotective effects mainly in rodent models in other neurodegenerative diseases, such as Parkinson’s disease, thanks to its antioxidative and anti-inflammatory properties [93,160,161]. In these cases, the effects were prevented by AM630, implying CB_2_R involvement [160,161,162].

Remarkably, research has shown that BCP might also be useful in substance abuse disorders. Treatment with the sesquiterpene decreased alcohol consumption and the conditioned-rewarding effect of ethanol in a murine model of voluntary alcohol intake [94]. The response was thought to be mediated via CB_2_R activation, since co-administration with AM630 abrogated BCP effects. On the other hand, high doses of BCP also prevented cocaine self-administration and relapse in rats in a CBR-independent manner [95]. However, in this study, PPARα and PPARγ agonists had a similar effect to BCP, and co-administration with PPARα or PPARγ antagonists reversed BCP-induced improvement. It is worth noting that substance use disorders and anxiety frequently co-occur [163], and BCP has also shown anxiolytic and antidepressant effects via CB_2_R activation [164].

Diverse studies have shown the antitumorigenic effects of cannabinoids and the ECS as a target, which have been reviewed recently [165]. This also includes the antiproliferative effects of CB_2_R [166,167,168] and PPARγ [35]. The dual CB_2_R/PPARγ ligands AJA, MHK, and BCP have proven to possess beneficial effects as antineoplastic agents in different experimental models. In several tumor cells, AJA inhibited tumor growth in a CB_2_R-dependent, CB_1_R-independent manner, and when tested in vivo, oral administration of AJA delayed the appearance and size of tumors in nude mice [96]. Moreover, AJA also had a modest improvement in survival [169]. It is worth noting that pro-inflammatory cytokines can promote microenvironments favoring tumor growth and invasiveness [170], and AJA can decrease the expression and secretion of these cytokines [132,134]. Meanwhile, MHK and BCP promote cell cycle arrest and subsequent apoptosis in several tumor cell lines by stimulating PPARγ activity and decreasing NF-κB DNA binding activity [97,98,171,172,173,174], which is a pattern already observed with other PPARγ ligands [175,176]. GW9662, a PPARγ antagonist, reversed the anti-cell growth and apoptotic effects of MHK and decreased phosphatase and tensin homolog (PTEN) expression [97,171], whereas AM630 prevented BCP antiproliferative response [98]. Other well-established CB_2_R/CB_1_R and CB_2_R ligands, such as WIN55,212-2 and JHW-015 respectively, have shown that their antineoplastic effects are also mediated through PPARγ activation [55,99].

Obesity and metabolic syndrome are associated to chronic low-grade inflammation. BCP has shown promising properties as a therapeutic agent in metabolic disorders. In preclinical studies, this sesquiterpene stimulates insulin secretion and sensitization [177,178], reduces glucose plasma levels and gastrointestinal absorption [179,180], decreases hyperlipidemia [179], promotes an anti-inflammatory and antioxidant environment, and protects against diabetic complications [139,181]. These effects are mediated through a combination of CB_2_R, PPARα, and PPARγ activation, and they have been recently reviewed elsewhere [103,182]. Other dual ligands, such as VCE-004.8, have also shown beneficial properties in animal models of obesity. Although this quinone shows a lower adipogenic profile than PPARγ full agonists, treatment with VCE-004.8 was able to significantly reduce weight gain, fat mass, and adiposity [100]. Moreover, VCE-004.8 decreased crown-like structures, which is a feature of macrophage infiltration in the adipose tissue of obese mice. The beneficial effects of VCE-004.8 on obesity might not be exclusively related to PPARγ activation. Research shows that beyond its effects on inflammation, CB_2_R is also involved in energy homeostasis and food intake [183]. Meanwhile, MHK displayed a low-to-moderate effect over body weight gain, fat mass, and adipocyte hypertrophy in mice [101]. However, MHK has protective effects in diabetes-related pathologies. In high-fat diet-fed mice, MHK prevents fibrosis, organ injury, and lipid accumulation in liver, heart, and kidney [101,184,185]. Remarkably, MHK significantly downregulated CB_1_R overexpression and increased the hepatic concentrations of several *N*-acetylethanolamines, including AEA, OEA, and PEA [186].

### 3.2. CB_2_R–PPARα

The co-activation of CB_2_R and PPARα has been much less explored, although some evidence suggests that simultaneously targeting both receptors may also be an interesting pharmacological option in some pathology.

Ulcerative colitis, one of the main types of inflammatory bowel disease, is characterized by chronic persistent inflammation in the gastrointestinal tract. Oral administration of PEA has been shown to protect against edema and mucosa erosion in a dinitrobenzene sulfonic acid (DNBS)-induced ulcerative colitis murine model [102]. Moreover, this acylethanolamide displayed anti-inflammatory effects that were counteracted by CB_2_R, GPR55, or PPARα antagonists. Since PEA does not bind directly to CB_2_R [62], authors suggest that PEA might modulate CB_2_R through the *entourage effect*. In fact, it is well established that PEA can inhibit AEA inactivation and thus activate CBRs indirectly [63]. 

As summarized herein, several CB_2_R-PPARγ dual ligands have shown therapeutic relevance in preclinical and/or clinical studies for the management of inflammation, cancer, and neurodegeneration. CB_2_R and PPARα concomitant actions remain to be further investigated in disease models.

## 4. FAAH–PPAR Modulation

The degradation of enzymes, uptake, and intracellular transport upregulate the level of endocannabinoids [13]. Some of the pathophysiological effects of the inhibitors of these proteins have been described to be mediated through PPARs (for an understanding review, see Pistis and O’Sullivan [40]). Thus, we will report here the most recent advances that have been especially focused on FAAH inhibitors and PPARα agonists from these last years. FAAH is a serine hydrolase responsible for the degradation of AEA [187,188], 2-AG [189], PEA, and OEA [39] among other fatty acid compounds. Dual FAAH/PPARs inhibitors associated with diseases presented below are reported in Table 1. 

The contribution of PPARα in the mechanism of action of FAAH inhibitors to reduce diverse nausea and abused-drug reward conditions has been evidenced in several preclinical studies [106,109]. Curiously, two FAAH inhibitors, URB597 and PF-3845 (Figure 1), showed different mechanisms of action to reduce acute nausea and anticipatory nausea in rodent models of conditioned [106]. PF-3845 suppressed acute nausea via PPARα, but not CB_1_R, whereas URB597 reduced anticipatory nausea via CB_1_R, but not PPARα. Despite the fact that these two nausea conditions have different molecular basis, the divergence between the two FAAH inhibitors may be due to a difference of selectivity and potency on FAAH as suggested by the authors, PF-3845 being more potent and selective than URB597 [106]. 

Dual FAAH/PPARα mechanisms of action were also investigated in opioid withdrawal. For instance, the aversive effects of acute naloxone-precipitated morphine withdrawal by oleoyl glycine (OlGly; Figure 1) was shown to be mediated by both CB_1_R and PPARα in a rat model [109]. OlGly has been described as an FAAH inhibitor and PPARα agonist in vitro but not as CBRs ligand CBRs [110]. These data suggest the indirect activation of CB_1_R via FAAH inhibition and subsequent elevation of AEA levels. Following with studies on OlGly in naloxone-precipitated morphine withdrawal in rodent models, Ayoub et al. [108] designed and synthesized a OlGly derivative, oleoyl alanine (HU595, Figure 1). In these experiments, the effect of HU595 was prevented by pre-treatment with either the PPARα antagonist MK886 or the CB_1_ antagonist AM251, suggesting a PPARα and CB_1_R mechanism of action. Interestingly, at equivalent doses, its effects lasted longer than for OlGly, suggesting that it could be due to the enhanced stability of HU595 to hydrolysis [108]. It is worth mentioning that the mechanism of action of OlGly in reward and withdrawal conditions cannot be generalized as dual. For instance, OlGly was effective in nicotine reward and withdrawal [110], and this effect was proposed to be mediated by PPARα rather than FAAH. This was based on the fact that in a nicotine-dependent mice-conditioned place preference paradigm, the PPAR-α antagonist GW6471 prevented the effect of OlGly, whereas the lack of activity at FAAH was based on in vitro luciferase assays in which OlGly showed low agonism [110].

While designing new N-acylethanolamine acid amidase (NAAA) inhibitors based on azetidine-nitrile pharmacophore, Malamas et al. [105] identified potent and selective dual NAAA–FAAH inhibitors in vitro. For instance, the inhibitor **52** (Figure 1) was found to be highly potent at NAAA and FAAH, showing negligible activity at MAGL, ABHD6, and cathepsin K. Such dual inhibitors represent an interesting strategy that combines two distinct anti-inflammatory molecular pathways. NAAA inhibition stimulates the PEA/PPAR-α anti-inflammatory signaling pathway, whereas FAAH inhibition activates the CBRs by increasing AEA levels. Using the same FAAH/NAAA dual strategy, Wu et al. [104] explored carmofur (Figure 1), a 5-fluorouracil used clinically as an antineoplastic drug, based on the fact that this molecule contains a urea group, which is a common group in inhibitors of FAAH and NAAH. In an acute lung injury model, the anti-inflammatory effect of carmofur was blocked by the PPARα antagonist MK886 and the CB_2_R antagonist SR144528. From a mechanistic point of view, carmofur did not bind either PPARα or CB_2_R in competitive binding assays, but it has been confirmed as FAAH and NAAA inhibitor in enzymatic assays. Thus, its mechanism of action has been suggested to be the through indirect activation of PPARα and CB_2_R [104].

Recently, Fotio et al. [107] studied the FAAH inhibition on tolerance to the anti-nociceptive effects of morphine in mice by monitoring nociceptive thresholds by the tail immersion assay. The FAAH inhibitor URB597 prevented the development of morphine tolerance without altering nociceptive thresholds. These effects were mediated by a mechanism involving CB_2_R and CB_1_R and PPARα [107]. The peripherally restricted FAAH inhibitor URB937 did not modified morphine tolerance in these assays, revealing a central mechanism. Therefore, combining a central acting FAAH inhibitor and an opioid could be an interesting strategy to multimodal analgesia, as suggested by these authors [107].

Similar to PPARγ agonists, FAAH inhibitors have been implicated in antiproliferative, pro-apoptotic, and cytotoxic effects on several cancerous cell (recently reviewed by Brunetti and colleagues [190]). In this context, a dual PPAR/FAAH strategy could offer synergistic effects for tumor management.

## 5. Other CBR–PPAR Modulatory Profiles

In addition to cannabinoid/PPAR compounds acting preferentially at one of the two CBRs, some of them exhibit potential therapeutic actions through either receptors or none of them, these last ones being included here from a structural point of view. An association of disease and cannabinoids presented below is reported in Table 1. 

### 5.1. CBR–PPARγ

Diverse phytocannabinoids, phytocannabinoid acids, and their quinone derivatives fall into this category. O’Sullivan et al. [74] determined the direct binding of CBD to PPARγ ligand binding domain performing fluorescence polarization assay, being CBD less potent than rosiglitazone and AJA. PPARγ and not CB_1_R/CB_2_R has been associated with CBD effects on a amyloid β-induced neurotoxicity animal model [112], and more recently on fear memory consolidation [111] and on blood–brain barrier (BBB) permeability following ischemia [119]. Caprioglio et al. [191] performed oxidative reactions on the phytocannabinoids CBD, cannabigerol (CBG), cannabichromene (CBC), and cannabinol (CBN) that led to hydroxyquinones (cannabinoquinoids: CBDQ (also known as HU-331), CBGQ, CBCQ, CBNQ respectively; Figure 1). All of them became PPAR-γ modulators, analyzing their PPARγ transcriptional activity in HEK-293T cells [191].

The phytocannabinoid cannabimovone (CBM; Figure 1) is a natural terpenoid structurally related to CBD that is devoid of significant affinity for either CB_1_R or CB_2_R [192], but it has been recently described as a PPARγ agonist that is able to stimulate insulin signaling in vitro [193].

In 2005, O’Sullivan and co-workers [120] provided strong evidence that THC is a PPARγ ligand that can produce PPARγ-mediated effects independent of direct CBRs activation. In their assays realized in rat aorta, THC induced time-dependent vasorelaxation through the increased bioavailability of nitric oxide and hydrogen peroxide production. This effect was unaffected by CB_1_R antagonism but inhibited by the PPARγ antagonist GW9662. THCA, the acidic precursor of THC, is a less efficacious agonist for both CB_1_R and CB_2_R than THC but displays higher potency at PPARγ than THC, as reviewed by Moreno-Sanz [194]. This activity at PPARγ has been confirmed in a model of Huntington’s disease where THCA showed neuroprotective activity through a PPARγ-dependent pathway [82].

The controlled oxidation of CBG, a minor component of *Cannabis sativa* but precursor of CBD and THC, led to the identification of the cannabigerol quinone VCE-003 (Figure 1) [118]. VCE-003 has been revealed to be a PPARγ partial agonist with no significant activity at CB_1_R and only moderate activity at CB_2_R. It has been explored successfully for its neuroinflammatory and neuroprotective properties in a murine model of multiple sclerosis [118]. A derivative of VCE-003, VCE-003.2 (also known as EHP-102; Figure 1), which does not exhibit activity at CBRs, binds and activates PPARγ transcriptional activity in competitive assays, as does its parent compound VCE-003. VCE-003.2 showed neuroprotective effects in a murine model of Huntington’s disease that was prevented by co-administration of the PPARγ antagonist T0070907 and in a murine model of Parkinson’s disease [114,115,116,117]. 

PPARγ plays a role in some of the potential therapeutic effects of the synthetic high-affinity CB_1_R and CB_2_R agonist WIN55,212-2. For instance, in a amyloid β-induced neuroinflammation model, WIN55,212-2 increases PPAR-γ signaling through CB_1_R, in addition to its direct action on both CB_1_R and CB_2_R [113]. It has also been described that the effects of WIN55,212-2 on behavioral sensitization induced by abused drugs were mediated through PPAR-γ [121].

As already mentioned, diverse endocannabinoids have been implicated in PPARs activation, and some of their mechanisms of action remain elusive. Studies such as the ones performed by Kaminski and co-workers [195] suggest that endocannabinoid metabolism may play an important role in the action of endocannabinoids at PPARs. In the mentioned studies, a COX-2 downstream metabolite of 2-AG, 15-desoxy-Δ^12,14^-prostaglandin-J_2_-glycerol ester (15d-PGJ_2_-G; Figure 1) which is a PPARγ activator, has been shown to be in part responsible for interleukin (IL)-2 suppression by 2-AG through effectively a PPARγ mechanism of action.

The direct action of cannabinoids on PPARs or their signaling has been widely reported, whereas PPAR agonists for activity at the CBRs have been much less studied. The phytochemical product magnolol (Figure 1), which is structurally related to MHK, is a PPARγ partial agonist that binds in a dimeric mode to the nuclear receptor [196]. Magnolol has been found to possess a wide variety of pharmacological actions that have been recently reviewed elsewhere [197]. Through PPARγ modulation, this neolignan exerts anti-inflammatory actions in animal models of acute lung injury [198], ulcerative colitis [199], and Alzheimer’s disease [200], as well as cardioprotective effects [201,202], and beneficial properties in metabolic disorders [203]. Interestingly, magnolol has also been found to act as a partial agonist of CB_1_R and CB_2_R receptors [204], and its derivatization has led to the design of novel synthetic cannabinoid ligands with improved affinity [205]. 

### 5.2. CBR–PPARα

In a computational approach, CBD was considered a poor ligand for the PPARα receptor type [206]. In the same study, the CBD derivatives cannabidiolic acid (CBDA; Figure 1), cannabigerolic acid (CBGA; Figure 1), and cannabigerol (CBG; Figure 1) were predicted dual PPARα/γ by performing MD simulations and docking using the crystallographic structures of PPARα and PPARγ, which was subsequently confirmed by luciferase reporter gene assays [206]. Recently, Rock et al. [125] showed that the anti-nausea effect of another phytocannabinoid, THCA, was blocked by a PPARα antagonist, whereas the effect of CBDA was mediated by the 5-HT_1a_. Thus, they proposed the co-administration of CBDA and THCA as a combined therapy for treating chemotherapy-induced nausea through different mechanisms. 

At PPARα, THC has been mentioned by Sun et al. [41] not binding to the purified PPARα ligand binding domain and not having effects on PPARα-driven transcriptional activity, whereas in studies by Takeda et al. [122], THC stimulated the activity of PPARα. This later showed that THC not only increases the expression of fatty acid 2-hydroxylase (FA2H) via the upregulation of PPARα expression in human breast cancer MDA-MB-231 cells but that THC also interferes with the PPARβ/δ-mediated inhibition of PPARα.

WIN55,212-2 was shown to increase PPAR-γ signaling, and it also displays PPARα-binding affinity with biphasic effects on PPARα gene-transcription activity [41]. This PPARα activation could explain the higher lipolytic effect of WIN55,212-2 compared to other cannabinoids. There are also evidence that the anticonvulsant effects of WIN55,212-2 assessed in mice could be mediated through CB_1_R and PPARα, since these effects are blocked by a combination of CB_1_R and PPARα antagonists [123].

The PPARα agonist fenofibrate binds both CB_1_R and CB_2_R with submicromolar affinity [207]. In [^35^S]-GTPγS binding assays, fenofibrate was characterized as a CB_2_R agonist, whereas at CB_1_R, it acts as partial agonist at low concentrations and negative allosteric modulator at high concentrations. In cell-based CB_2_R/β-arrestin assays, fenofibrate was also identified as a CB_2_R agonist [208]. Thus, this PPARα activator can be considered a multitarget agent due to its possible activity at CBRs in addition to its primary PPAR target.

The acylethanolamides OEA and PEA are structurally related to the endogenous cannabinoid AEA with which they compete for the endocannabinoid-metabolizing enzyme, FAAH. In luciferase reporter gene assays and LBD of human PPARs, OEA binds PPAR-α with high affinity and PPARβ/δ but not PPARγ, whereas PEA only binds PPAR-α [56,209]. They also act at TRPV1 and GPR55 [13]. As already commented in this review, OEA and PEA do not bind CBRs; however, some of their activities have been suggested to be mediated through CBRs.

In the context of a dual PPAR/CBR strategy, it is worth mentioning the randomized, placebo-controlled, double-blind controlled trial realized on healthy human subjects in which a state of increased gut permeability was induced by aspirin [124]. In this clinical study, CBD and PEA could reduce human inflamed gastrointestinal permeability in vivo, suggesting the potential of these compounds for clinical use in inflammation bowel diseases.

As detailed in this section, a range of cannabinoids exert their therapeutic effects, acting preferentially via PPAR. On the other hand, specific compounds mediate their beneficial actions through both CB_1_R and CB_2_R along with a PPAR subtype. These pharmacological profiles are also worth exploring due to their anti-inflammatory, neuroprotective, or antitumoral properties. 

Functional profiles of all these multitarget cannabinoids are summarized in Table 2.

## 6. Conclusions and Future Perspectives

The ECS is involved in a variety of multifactorial pathologies including neurodegenerative diseases, cancer, and metabolic syndrome. In this context, multitargeting strategies offer better hopes for an effective treatment due to the synergistic therapeutic effects triggered upon the modulation of diverse signaling pathways. 

Molecular targets of the cannabinoids include the cannabinoid receptors CB_1_R and CB_2_R, orphan GPCRs, such as GPR18 and GPR55, ionotropic receptors such as GlyRs or GABA receptors, TRP channels including TRPV1-4, TRPA1, and TRPM8, and the PPAR family of nuclear receptors. The intrinsic complexity of the ECS along with variations associated to each disease phenotype led to diverse therapeutic options targeting this signaling system. This review provides a summary of reported molecules mainly exerting therapeutic actions via PPARs and the cannabinoid receptors and/or FAAH. In this context, promising dual strategies are synergistic CB_1_R blockage-PPARα activation, which offers therapeutic opportunities for the treatment of obesity or the coactivation of CB_2_R and PPARγ, which has shown great efficacy in the management of cancer or fibrosis.

Molecules exhibiting therapeutic actions mediated by CBR/PPAR targeting include endogenous, phytogenic, and synthetic compounds (the functional profile of these compounds has been summarized in Table 2). For instance, the endogenous *N*-acetylethanolamine PEA directly modulates PPARα and indirectly modulates the CBRs exerting antiepileptic, analgesic, or anti-inflammatory effects in different in vivo models. Phytogenic molecules including CBD, MHK, or BCP have also shown therapeutic properties mediated through CBR and PPAR responses. It is also worth highlighting the promising potential of cannabinoid acids. While the natural THC analogue THCA displays anti-arthritis activity through CB_1_R and PPARγ pathways, the synthetic THC acid derivative AJA exhibits antitumoral and anti-inflammatory effects via CB_2_R and PPARγ activation. The promising pharmacological potential of the latter has led it into clinical trials for chronic inflammatory diseases including systemic sclerosis, cystic fibrosis, dermatomyositis, and systemic lupus erythematosus [146,169,215]. The most remarkable synthetic compounds with this dual profile are the cannabinoid quinones. In fact, the cannabidiol quinone derivative developed by Emerald Health Pharmaceuticals, VCE-004.8, has entered clinical trials for its anti-inflammatory and neuroprotective properties [147]. Dual FAAH–PPAR activity has also been highlighted for its multitarget therapeutic value. Indeed, molecules acting as FAAH inhibitors and PPAR activators have shown beneficial actions for the treatment of pain, cancer, or inflammation.

Tridimensional structures of the cannabinoid and nuclear receptors and the fatty acid enzyme FAAH have been recently solved. While numerous crystallographic studies have been reported for all PPAR types, CB_1_R and CB_2_R have not been elucidated until the past few years. PPARα and γ have been solved in complex with a wide range of ligands [216,217,218,219]. However, thus far, the only reported PPAR structure bound to a cannabinoid is the AJA–PPARγ complex [210]. In what concerns the cannabinoid receptors, crystal structures of the active and inactive states [15,16,17] and more recently cryo-EM CB_1_R–G_i_ and CB_2_R–G_i_ structures in complex with agonists [19,21,22] have helped the understanding of their activation and signaling mechanism. Moreover, a high-resolution crystal structure of FAAH bound to its inhibitor URB597 has also been reported [220]. All these structures may help understanding the molecular basis of cannabinoid dual targeting and guide future drug design.

It is important to highlight that compounds exhibiting CBR/PPAR dual profile might be able to reach cell membrane and nuclear receptors. Cannabinoids are lipophilic molecules that reach their CBR binding site upon diffusion through the lipid bilayer from the extracellular milieu. The lipid GPCR structures reported in the last years have shed light into a better understanding of how cannabinoids reach their targets [15,16,17,19,21,22]. Portals in the transmembrane helices may facilitate their access to the orthosteric binding site while allosteric sites have been found at the receptor interface. However, it is not fully determined how cannabinoids access the PPAR binding crevice. On the basis of reviewed studies, O’Sullivan [31,39,40] suggested different mechanisms of action: (i) Activation of the CBRs by cannabinoids induces signaling cascades that indirectly activate PPAR; (ii) Cannabinoids can bind directly to PPARs; (iii) Cannabinoids can be transported to PPARs by fatty acid-binding proteins (FABPs) s; (iv) Cannabinoid metabolites can be the ones that activate PPARs (Figure 2). These mechanisms may vary depending on each molecule; however, further mechanistic studies remain to be done in order to determine cannabinoid–PPAR interactions. 

In addition to well-known unbiased modulatory profiles, functionally selective or biased agonists have recently emerged as a promising pharmacological approach in GPCRs [221]. Increasing evidence has shown that β-arrestin 1 and β-arrestin 2 can also recruit signaling mediators to the activated receptors, thus eliciting signaling cascades beyond G protein-mediated signals [221]. Functional selectivity at CBRs has been intensively studied in the past few years [222,223,224]. However, how biased modulators of CBRs toward one or the other signaling pathway affect these CBR/PPAR dual strategies remains to be characterized. 

Interestingly, it has been reported that after the GPCR activation of specific receptors (such as the κ and δ opioid receptors [225]), β-arrestin 1, but not β-arrestin 2, can be actively transported to the nucleus [226]. Nuclear translocation correlates to an increased gene transcription of cell proliferation regulating genes, and presumably, histone modification and chromatin remodeling [225]. Moreover, β-arrestin 1 has been shown to directly interact with the LBD of both PPARα and PPARγ and modulate their transcriptional activity [227,228]. Although there has been no evidence yet in this regard, research is needed to determine if cannabinoid receptor activation could modulate PPAR transcriptional activity via β-arrestin 1 nuclear translocation and whether biased cannabinoid ligands elicit different responses through this mechanism. In addition to CBR functional selectivity, the therapeutic relevance of CBR allosteric modulation/PPAR activation should be further explored under diverse physiopathological conditions.

Extensive research supports the relevance of CBR–PPAR dual targeting in the context of a broad range of pathologies. In fact, multitarget drugs can offer an improved therapeutic profile in complex diseases, as already occurs in multifactorial disorders such as cancer, neurodegenerative diseases, or psychiatric disorders [229]. In this sense, CB_2_R and PPARγ modulators are at the forefront of multitarget cannabinoid-related drugs. Compounds such as AJA or EHP-101 have successfully reached clinical stages where they are being evaluated in autoimmune disorders, including systemic sclerosis. As a result of their polypharmacological profile, these drugs can offer therapeutic advances over the current combinatorial strategies, not only because of the synergistic effect that their molecular targets exert but also by improving the pharmacokinetic profile and reducing the risk of toxicity and drug–drug interactions when multiple drugs are administered. 

While the studies reviewed herein have greatly enhanced our understanding of the cannabinoids/PPARs interactions, there are important challenges that remain to be investigated. With the exception of the aforementioned CB_2_R/PPARγ compounds, all the data summarized herein comes from animal models; therefore, the translational potential of these multitarget drugs remains to be determined in clinical trials. The design of selective and potent dual ligands may help further understanding the relevance of multitargeting approaches within the ECS.

## Figures and Tables

**Table 1:** Summary of cannabinoids exerting their therapeutic properties through CBR/fatty acid amide hydrolase (FAAH) and PPAR associated by target and disease.

Targets Involved	Pathologies	Compounds	Ref.
CB_1_R–PPARα	Metabolic syndrome	OLHHA	[48,49,50]
		Rimonabant fibrate **2**	[52]
		AM6545	[53]
		OEA + SR144716A	[54]
	Alcohol use disorder	OLHHA	[51]
	Epilepsy	PEA	[61]
	Nociception	PEA	[65]
		AEA + GW7647	[66]
CB_1_R–PPARγ	Cancer	Chromenopyrazoledione **4**	[71]
		CBD	[73]
	Obesity	EMI	[77]
	Arthritis	THCA	[81]
CB_2_R–PPARγ	Arthritis	AJA	[83]
		BCP	[84]
	Fibrosis	AJA	[85]
		VCE-004.3	[86]
		VCE-004.8	[87,88]
	Multiple sclerosis	BCP	[89]
		VCE-004.8	[90]
	Alzheimer’s disease	MHK	[91]
		BCP	[92]
	Parkinson’s disease	BCP	[93]
	Substance abuse	BCP	[94,95]
	Cancer	AJA	[96]
		MHK	[97]
		BCP	[98]
		WIN55,212-2	[99]
		JHW-015	[55]
	Metabolic dysfunction	VCE-004.8	[100]
		MHK	[101]
CB_2_R–PPARα	Ulcerative colitis	PEA	[102]
CB_2_R–PPARα/γ	Metabolic dysfunction	BCP	[103]
FAAH–PPARα	Inflammation	Carmofur ^‡‡^	[104]
		Azetidine-nitrile **52**	[105]
	Nausea	PF-3845	[106]
	Opioid tolerance	URB597 ^‡‡‡^	[107]
	Opioid withdrawal	HU595 ^‡^	[108]
		OlGly ^‡^	[109]
	Nicotine withdrawal	OlGly ^‡^	[110]
CBR–PPARγ	Memory	CBD *	[111]
	Alzheimer’s disease	CBD *	[112]
		WIN55,212-2 **	[113]
	Huntington’s disease	THCA*	[82]
		VCE-003.2 *	[114,115]
	Parkinson’s disease	VCE-003.2 *	[116,117]
	Multiple sclerosis	VCE-003 *	[118]
	BBB permeability following ischemia	CBD *	[119]
	Vasorelaxation	CBD *	[74]
		THC *	[120]
	Addiction	WIN55,212-2 *	[121]
CBR–PPARα	Cancer	THC *	[122]
	Anticonvulsant	WIN55,212-2 **	[123]
	Inflammation	PEA *	[124]
	Nausea	THCA *	[125]

^‡^ FAAH inhibitors that mediate therapeutic effects via PPAR and CB1R. ^‡‡^ FAAH inhibitors that mediate therapeutic effects via PPAR and CB2R. ^‡‡‡^ FAAH inhibitors that mediate therapeutic effects via PPAR and both CBRs. * Cannabinoids that mediate therapeutic effects via PPAR. ** Cannabinoids that mediate therapeutic effects via PPAR and both CBRs.

**Table 2:** Multitarget functional profile of cannabinoids and FAAH inhibitors acting at PPARs in the context of the revised pathologies.

Compounds	Targets					References
	CB1R	CB2R	PPARα	PPARγ	FAAH	
OEA			+			[56]
PEA	[+]	[+]	+	+		[61,65,102]
OLHHA	-		+			[48,50,51]
OlGly	[+]	NE	+		-	[109,110]
HU595	[+]		+		-	[106,108]
Magnolol	+	+	+	+		[159,197,204]
MHK		*	+	+		[159,186]
EMI	UR			UR		[77]
BCP	NE	+	+	[+]		[84,139,140]
CBD	*	*		+		[74,111,112,118,119,206]
CBDA			+	+		[206]
THC	+	+	*	+		[41,74,120,122]
THCA	+ ***	-	[+]	+		[81,82,125,194]
AJA		+	NE	+		[210,211]
CBM	NE	NE	+	+		[193]
CBG			+	+		[74,191,206]
CBGA			+	+		[206]
Chromenopyrazole 4	+			+		[71]
VCE-004.8	NE	+		+		[212]
VCE-004.3	-	+		+		[86]
VCE-003; CBGQ	NE	+		+		[118,191]
VCE-003.2	NE	NE		+		[114,115,116,117]
CBDQ; HU-331	NE	NE		+		[191]
CBCQ				+		[191]
CBNQ				+		[191]
Fenofibrate	+	+	+			[207]
AM6545	- **		[+]			[53]
Rimonabant fibrate 2	-		+			[52]
WIN55,212	+	+	+	[+]		[41,99,113,123]
JHW-015		+		[+]		[55]
URB597	[+]	[+]	[+]		-	[106,107,213]
PF-3845	[+]		[+]		-	[107]
Carmofur		[+]	[+]		-	[104]
Azetidine-nitrile 52	[+]	[+]	[+]		-	[105]

+ Agonist; - Antagonist/inverse agonist; [+]: indirect activation; NE: no effect; UR: upregulation. * divergences among reports (for CBD see ref. [214]; for MHK see [158]; for THC [41,122]); ** Peripheral modulation; *** Allosteric modulation.

## Data Availability

Not applicable.

## Abbreviations

15d-PGJ_2_-G	15-Desoxy-Δ12,14-prostaglandin-J2-glycerol ester
2-AG	2-Arachinoylglycerol
5-HT_1a_	Serotonin 1A receptor
ABHD	α/β Hydrolase domain
AEA	*N*-Arachidonoyl-ethanolamine
AJA	Ajulemic acid
BBB	Blood brain barrier
BCP	β-Caryophyllene
CB_1_R	Cannabinoid receptor type 1
CB_2_R	Cannabinoid receptor type 2
CBC	Cannabichromene
CBCQ	Cannabichromenquinone
CBD	Cannabidiol
CBDA	Cannabidiolic acid
CBDQ	Cannabidiol hydroxyquinone
CBG	Cannabigerol
CBGA	Cannabigerolic acid
CBGQ	Cannabigeroquinone
CBM	Cannabimovone
CBN	Cannabinol
CBNQ	Cannabinolquinone
CBR	Cannabinoid receptor
Cryo-EM	Cryo electron microscopy
DGL	Diacylglycerol lipase
DIO	Diet-induced obese
ECS	Endocannabinoid System
EMI	Mangifera indica
EMT	Endocannabinoid membrane transporter
FA2H	Fatty acid 2-hydroxylase
FAAH	Fatty acid amide hydrolase
FABP	Fatty acid binding protein
GlyR	Glycine receptor
GPCR	G-Protein coupled receptor
LBD	Ligand binding domain
MAGL	Monoacylglycerol lipase
MD	Molecular dynamic
MHK	4′-*O*-Methylhonokiol
NAAA	*N*-Acylethanolamine acid amidase
NAFLD	Non-alcoholic fatty liver disease
NAPE-PLD	*N*-Acyl phosphatidylethanolamine-specific phospholipase D
OEA	Oleoylethanolamide
OlGly	Oleoyl glycine
OLHHA	Oleic acid–dihydroxyamphetamine
PEA	Palmitoylethanolamide
PPAR	Peroxisome proliferator-activated receptors
PPRE	Peroxisome proliferator response element
THC	Δ^9^-Tetrahydrocannabinol
THCA	Δ^9^-Tetrahydrocannabinolic acid
TRP	Transient receptor potential
TRPM	Transient receptor potential melastatin
TZD	Thiazolidinedione
T2DM	Type 2 diabetes mellitus

## References

[1] Morphy R., Kay C., Rankovic Z. (2004). From magic bullets to designed multiple ligands. Drug Discov. Today.

[2] Anighoro A., Bajorath J., Rastelli G. (2014). Polypharmacology: Challenges and Opportunities in Drug Discovery. J. Med. Chem..

[3] Proschak E., Stark H., Merk D. (2019). Polypharmacology by Design: A Medicinal Chemist’s Perspective on Multitargeting Compounds. J. Med. Chem..

[4] Costantino L., Barlocco D. (2012). Designed multiple ligands: Basic research vs. clinical outcomes. Curr. Med. Chem..

[5] Naveja J.J., González F.I.S., Cruz N.S., Franco J.L.M. (2018). Cheminformatics Approaches to Study Drug Polypharmacology. Methods in Pharmacology and Toxicology.

[6] Pertwee R.G. (2014). Handbook of Cannabis.

[7] Matsuda L.A., Lolait S.J., Brownstein M.J., Young A.C., Bonner T.I. (1990). Structure of a cannabinoid receptor and functional expression of the cloned cDNA. Nature.

[8] Devane W., Hanus L., Breuer A., Pertwee R., Stevenson L., Griffin G., Gibson D., Mandelbaum A., Etinger A., Mechoulam R. (1992). Isolation and structure of a brain constituent that binds to the cannabinoid receptor. Science.

[9] Mechoulam R., Shabat S.B., Hanus L., Ligumsky M., Kaminski N.E., Schatz R., Gopher A., Almog S., Martin B.R., Compton D.R. (1995). Identification of an endogenous 2-monoglyceride, present in canine gut, that binds to cannabinoid receptors. Biochem. Pharmacol..

[10] Simcocks A.C., Jenkin K.A., O’Keefe L., Samuel C.S., Mathai M.L., McAinch A.J., Hryciw D.H. (2019). Atypical cannabinoid ligands O-1602 and O-1918 administered chronically in diet-induced obesity. Endocr. Connect..

[11] Munro S., Thomas K.L., Shaar M.A. (1993). Molecular characterization of a peripheral receptor for cannabinoids. Nature.

[12] Mechoulam R., Hanus L.O., Pertwee R., Howlett A.C. (2014). Early phytocannabinoid chemistry to endocannabinoids and beyond. Nat. Rev. Neurosci..

[13] di Marzo V. (2018). New approaches and challenges to targeting the endocannabinoid system. Nat. Rev. Drug Discov..

[14] Morales P., Jagerovic N. (2020). Novel approaches and current challenges with targeting the endocannabinoid system. Expert Opin. Drug Discov..

[15] Shao Z., Yin J., Chapman K., Grzemska M., Clark L., Wang J., Rosenbaum D.M. (2016). High-resolution crystal structure of the human CB1 cannabinoid receptor. Nature.

[16] Hua T., Vemuri K., Pu M., Qu L., Han G.W., Wu Y., Zhao S., Shui W., Li S., Korde A. (2016). Crystal Structure of the Human Cannabinoid Receptor CB1. Cell.

[17] Li X., Hua T., Vemuri K., Ho J.H., Wu Y., Wu L., Popov P., Benchama O., Zvonok N., Locke K. (2019). Crystal Structure of the Human Cannabinoid Receptor CB2. Cell.

[18] Hua T., Vemuri K., Nikas S.P., Laprairie R.B., Wu Y., Qu L., Pu M., Korde A., Jiang S., Ho J.-H. (2017). Crystal structures of agonist-bound human cannabinoid receptor CB1. Nature.

[19] Hua T., Li X., Wu L., Iliopoulos-Tsoutsouvas C., Wang Y., Wu M., Shen L., Johnston C.A., Nikas S.P., Song F. (2020). Activation and Signaling Mechanism Revealed by Cannabinoid Receptor-Gi Complex Structures. Cell.

[20] Shao Z., Yan W., Chapman K., Ramesh K., Ferrell A.J., Yin J., Wang X., Xu Q., Rosenbaum D.M. (2019). Structure of an allosteric modulator bound to the CB1 cannabinoid receptor. Nat. Chem. Biol..

[21] Krishna Kumar K., Shalev-Benami M., Robertson M.J., Hu H., Banister S.D., Hollingsworth S.A., Latorraca N.R., Kato H.E., Hilger D., Maeda S. (2019). Structure of a Signaling Cannabinoid Receptor 1-G Protein Complex. Cell.

[22] Xing C., Zhuang Y., Xing C., Zhuang Y., Xu T., Feng Z., Zhou X.E., Chen M., Wang L. (2020). Cryo-EM Structure of the Human Cannabinoid Receptor CB2-G i Signaling Complex. Cell.

[23] Li X., Shen L., Hua T., Liu Z.J. (2020). Structural and Functional Insights into Cannabinoid Receptors. Trends Pharmacol. Sci..

[24] Long J.Z., Nomura D.K., Vann R.E., Walentiny D.M., Booker L., Jin X., Burston J.J., Sim-selley L.J., Lichtman A.H., Wiley J.L. (2009). Dual blockade of FAAH and MAGL identifies behavioral processes regulated by endocannabinoid crosstalk in vivo. Proc. Natl. Acad. Sci. USA.

[25] Abood M., Alexander S., Barth F., Bonner T., Bradshaw H., Cabral G., Casellas P., Cravatt B., Devane W., di Marzo V. (2019). Cannabinoid Receptors (Version 2019.4) in the IUPHAR/BPS Guide to Pharmacology Database. IUPHAR/BPS Guide to Pharmacology CITE. https://www.guidetopharmacology.org/GRAC/FamilyDisplayForward?familyId=13.

[26] Morales P., Reggio P.H. (2017). An Update on Non-CB1, Non-CB2 Cannabinoid Related G-Protein-Coupled Receptors. Cannabis Cannabinoid Res..

[27] Muller C., Morales P., Reggio P.H. (2019). Cannabinoid Ligands Targeting TRP Channels. Front. Mol. Neurosci..

[28] Zhang L. (2016). Therapeutic Potential of Nonpsychoactive Cannabinoids by Targeting at Glycine Receptors. Cannabinoids in Health and Disease.

[29] Xiong W., Cheng K., Cui T., Godlewski G., Rice K.C., Xu Y., Zhang L. (2011). Cannabinoid potentiation of glycine receptors contributes to cannabis-induced analgesia. Nat. Chem. Biol..

[30] Aso E., Fernández-Dueñas V., López-Cano M., Taura J., Watanabe M., Ferrer I., Luján R., Ciruela F. (2019). Adenosine A2A-Cannabinoid CB1 Receptor Heteromers in the Hippocampus: Cannabidiol Blunts Δ9-Tetrahydrocannabinol-Induced Cognitive Impairment. Mol. Neurobiol..

[31] O’Sullivan S.E., Kendall D.A. (2010). Cannabinoid activation of peroxisome proliferator-activated receptors: Potential for modulation of inflammatory disease. Immunobiology.

[32] Bagher A.M., Laprairie R.B., Kelly M.E.M., Wright E.M.D. (2016). Antagonism of Dopamine Receptor 2 Long Affects Cannabinoid Receptor 1 Signaling in a Cell Culture Model of Striatal Medium Spiny Projection Neurons. Mol. Pharmacol..

[33] Sierra S., Gupta A., Gomes I., Fowkes M., Ram A., Bobeck E.N., Devi L.A. (2019). Targeting Cannabinoid 1 and Delta Opioid Receptor Heteromers Alleviates Chemotherapy-Induced Neuropathic Pain. ACS Pharmacol. Transl. Sci..

[34] Franco R., Villa M., Morales P., Resina I.R., Rodríguez A.G., Jiménez J., Jagerovic N., Orgado J.M., Navarro G. (2019). Increased expression of cannabinoid CB2 and serotonin 5-HT1A heteroreceptor complexes in a model of newborn hypoxic-ischemic brain damage. Neuropharmacology.

[35] Yousefnia S., Momenzadeh S., Forootan F.S., Ghaedi K., Esfahani M.H.N. (2018). The influence of peroxisome proliferator-activated receptor γ (PPARγ) ligands on cancer cell tumorigenicity. Gene.

[36] Decara J., Rivera P., Gambero A.J.L., Serrano A., Pavón F.J., Baixeras E., de Fonseca F.R., Suárez J. (2020). Peroxisome Proliferator-Activated Receptors: Experimental Targeting for the Treatment of Inflammatory Bowel Diseases. Front. Pharmacol..

[37] Berger J., Moller D.E. (2002). The mechanisms of action of PPARs. Annu. Rev. Med..

[38] Takada I., Makishima M. (2020). Peroxisome proliferator-activated receptor agonists and antagonists: A patent review (2014-present). Expert Opin. Ther. Pat..

[39] O’Sullivan S.E. (2007). Cannabinoids go nuclear: Evidence for activation of peroxisome proliferator-activated receptors. Br. J. Pharmacol..

[40] Pistis M., O’Sullivan S.E. (2017). The Role of Nuclear Hormone Receptors in Cannabinoid Function.

[41] Sun Y., Alexander S.P.H., Garle M.J., Gibson C.L., Hewitt K., Murphy S.P., Kendall D.A., Bennett A.J. (2007). Cannabinoid activation of PPAR alpha; a novel neuroprotective mechanism. Br. J. Pharmacol..

[42] O’Sullivan S.E. (2013). Cannabinoid activation of peroxisome proliferator-activated receptors: An update and review of the physiological relevance. Wiley Interdiscip. Rev. Membr. Transp. Signal..

[43] Yadav M.R., Murumkar P.R. (2018). Advances in patented CB1 receptor antagonists for obesity. Pharm. Pat. Anal..

[44] Murphy T., Foll B.L. (2020). Targeting the endocannabinoid CB1 receptor to treat body weight disorders: A preclinical and clinical review of the therapeutic potential of past and present CB1 drugs. Biomolecules.

[45] Jagerovic N., Fernandez-fernandez C., Goya P. (2008). CB1 Cannabinoid Antagonists: Structure-Activity Relationships and Potential Therapeutic Applications. Curr. Top. Med. Chem..

[46] Stienstra R., Duval C., Müller M., Kersten S. (2007). PPARs, obesity, and inflammation. PPAR Res..

[47] Yoon M. (2009). The role of PPARα in lipid metabolism and obesity: Focusing on the effects of estrogen on PPARα actions. Pharmacol. Res..

[48] Almeida B., Joglar J., Rojas M.J.L., Decara J.M., Silva F.J.B., González M.M., Fitó M., Cuevas M.R., Farré M., Covas M.I. (2010). Synthesis of fatty acid amides of catechol metabolites that exhibit antiobesity properties. ChemMedChem.

[49] de la Torre Fornell R., Albadalejo M.F., Planells M.I.C., Colomert M.F., Cotrim B.A., de Fonseca F.R., del Olmo J.M.D., González M.M., Cuevas M.R., Amargo J.J. (2011). Fatty Acid Amide Derivatives with Amphetamines for the Treatment of Eating. Disorders. Patent.

[50] Decara J.M., Pavón F.J., Suárez J., Cuevas M.R., Baixeras E., Vázquez M., Rivera P., Gavito A.L., Almeida B., Joglar J. (2015). Treatment with a novel oleic-acid-dihydroxyamphetamine conjugation ameliorates non-alcoholic fatty liver disease in obese Zucker rats. DMM Dis. Model. Mech..

[51] Alen F., Decara J., Brunori G., You Z.B., Bühler K.M., Moreno J.A.L., Cippitelli A., Pavon F.J., Suárez J., Gardner E.L. (2018). PPARα/CB1 receptor dual ligands as a novel therapy for alcohol use disorder: Evaluation of a novel oleic acid conjugate in preclinical rat models. Biochem. Pharmacol..

[52] Fernández R.P., Fresno N., González M.M., Elguero J., Decara J., Girón R., Rodríguez-Álvarez A., Martín M.I., Rodríguez De Fonseca F., Goya P. (2011). Discovery of potent dual PPARα agonists/CB1 ligands. ACS Med. Chem. Lett..

[53] Azar S., Udi S., Drori A., Hadar R., Nemerovski A., Vemuri K.V., Miller M., Rofe D.S., Arad Y., Wahnon D.G. (2020). Reversal of Diet-induced Hepatic Steatosis by Peripheral CB1 Receptor Blockade in Mice is p53/miRNA-22/SIRT1/PPARα Dependent. Mol. Metab..

[54] Serrano A., del Arco I., Pavón F.J., Macías M., Valero V.P., de Fonseca F.R. (2008). The cannabinoid CB1 receptor antagonist SR141716A (Rimonabant) enhances the metabolic benefits of long-term treatment with oleoylethanolamide in Zucker rats. Neuropharmacology.

[55] Vara D., Morell C., Henche N.R., Laviada I.D. (2013). Involvement of PPARγ in the antitumoral action of cannabinoids on hepatocellular carcinoma. Cell Death Dis..

[56] Fu J., Gaetani S., Oveisi F., Verme J.L., Serrano A., de Fonseca F.R., Rosengarth A., Luecke H., di Giacomo B., Tarzia G. (2003). Oleylethanolamide regulates feeding and body weight through activation of the nuclear receptor PPAR-α. Nature.

[57] Panikashvili D., Mechoulam R., Beni S.M., Alexandrovich A., Shohami E. (2005). CB1 cannabinoid receptors are involved in neuroprotection via NF-κB inhibition. J. Cereb. Blood Flow Metab..

[58] Van der Stelt M., di Marzo V. (2005). Cannabinoid receptors and their role in neuroprotection. Neuro. Mol. Med..

[59] Wójtowicz S., Strosznajder A.K., Jeżyna M., Strosznajder J.B. (2020). The Novel Role of PPAR Alpha in the Brain: Promising Target in Therapy of Alzheimer’s Disease and Other Neurodegenerative Disorders. Neurochem. Res..

[60] Bordet R., Gelé P., Duriez P., Fruchart J.C. (2006). PPARs: A new target for neuroprotection. J. Neurol. Neurosurg. Psychiatry.

[61] Citraro R., Russo E., Scicchitano F., van Rijn C.M., Cosco D., Avagliano C., Russo R., D’Agostino G., Petrosino S., Guida F. (2013). Antiepileptic action of N-palmitoylethanolamine through CB1 and PPAR-α receptor activation in a genetic model of absence epilepsy. Neuropharmacology.

[62] Lambert D.M., Di Marzo V. (1999). The palmitoylethanolamide and oleamide enigmas: Are these two fatty acid amides cannabimimetic?. Curr. Med. Chem..

[63] Ho W.S.V., Barrett D.A., Randall M.D. (2008). “Entourage” effects of N-palmitoylethanolamide and N-oleoylethanolamide on vasorelaxation to anandamide occur through TRPV1 receptors. Br. J. Pharmacol..

[64] Jonsson K.O., Vandevoorde S., Lambert D.M., Tiger G., Fowler C.J. (2001). Effects of homologues and analogues of palmitoylethanolamide upon the inactivation of the endocannabinoid anandamide. Br. J. Pharmacol..

[65] Alsalem M., Haddad M., Aldossary S.A., Kalbouneh H., Altarifi A., Jaffal S.M., Abbas M.A., Aldaoud N., El-Salem K. (2019). Role of cannabinoid receptor 1 and the peroxisome proliferator-activated receptor α in mediating anti-nociceptive effects of synthetic cannabinoids and a cannabinoid-like compound. Inflammopharmacology.

[66] Russo R., Verme J.L., Rana G.L., D’Agostino G., Sasso O., Calignano A., Piomelli D. (2007). Synergistic antinociception by the cannabinoid receptor agonist anandamide and the PPAR-α receptor agonist GW7647. Eur. J. Pharmacol..

[67] O’Sullivan S.E. (2016). An update on peroxisome proliferator-activated receptor (PPAR) activation by cannabinoids. Br. J. Pharmacol..

[68] Ramer R., Schwarz R., Hinz B. (2019). Modulation of the endocannabinoid system as a potential anticancer strategy. Front. Pharmacol..

[69] Dariš B., Verboten M.T., Knez Ž., Ferk P. (2019). Cannabinoids in cancer treatment: Therapeutic potential and legislation. Bosn. J. Basic Med. Sci..

[70] Laezza C., Pagano C., Navarra G., Pastorino O., Proto M.C., Fiore D., Piscopo C., Gazzerro P., Bifulco M. (2020). The endocannabinoid system: A target for cancer treatment. Int. J. Mol. Sci..

[71] Morales P., Vara D., Cañas M.G., Zúñiga M.C., Azar C.O., Goya P., Ruiz J.F., Laviada I.D., Jagerovic N. (2013). Synthetic cannabinoid quinones: Preparation, in vitro antiproliferative effects and in vivo prostate antitumor activity. Eur. J. Med. Chem..

[72] Seltzer E.S., Watters A.K., Mackenzie D., Granat L.M., Zhang D. (2020). Cannabidiol (CBD) as a promising anti-cancer drug. Cancers.

[73] Aviello G., Romano B., Borrelli F., Capasso R., Gallo L., Piscitelli F., Di Marzo V., Izzo A.A. (2012). Chemopreventive effect of the non-psychotropic phytocannabinoid cannabidiol on experimental colon cancer. J. Mol. Med..

[74] O’Sullivan S.E., Sun Y., Bennett A.J., Randall M.D., Kendall D.A. (2009). Time-dependent vascular actions of cannabidiol in the rat aorta. Eur. J. Pharmacol..

[75] McPartland J.M., Glass M., Pertwee R.G. (2007). Meta-analysis of cannabinoid ligand binding affinity and receptor distribution: Interspecies differences. Br. J. Pharmacol..

[76] Ramer R., Heinemann K., Merkord J., Rohde H., Salamon A., Linnebacher M., Hinz B. (2013). COX-2 and PPAR-γ confer cannabidiol-induced apoptosis of human lung cancer cells. Mol. Cancer Ther..

[77] Brito L.F., Gontijo D.C., Toledo R.C.L., Barcelos R.M., de Oliveira A.B., Brandão G.C., de Sousa L.P., Ribeiro S.M.R., Leite J.P.V., Fietto L.G. (2019). Mangifera indica leaves extract and mangiferin modulate CB1 and PPARγ receptors and others markers associated with obesity. J. Funct. Foods.

[78] Fahmi H., Pelletier J.M., Pelletier J.-P., Kapoor M. (2011). Peroxisome proliferator-activated receptor gamma in osteoarthritis. Mod. Rheumatol..

[79] Marder W., Khalatbari S., Myles J.D., Hench R., Lustig S., Yalavarthi S., Parameswaran A., Brook R.D., Kaplan M.J. (2013). The peroxisome proliferator activated receptor-γ pioglitazone improves vascular function and decreases disease activity in patients with rheumatoid arthritis. J. Am. Heart Assoc..

[80] Lowin T., Straub R.H. (2015). Cannabinoid-based drugs targeting CB1 and TRPV1, the sympathetic nervous system, and arthritis. Arthritis Res. Ther..

[81] Palomares B., Rodriguez M.G., Consuegra C.G., Cañas M.G., Saen-oon S., Soliva R., Collado J.A., Ruiz J.F., Morello G., Calzado M.A. (2020). Δ9-Tetrahydrocannabinolic acid alliviates collagen-induced arthritis: Role of PPARγ and CB1 receptors. Br. J. Pharmacol..

[82] Nadal X., del Río C., Casano S., Palomares B., Vera C.F., Navarrete C., Carnerero C.S., Cantarero I., Bellido M.L., Meyer S. (2017). Tetrahydrocannabinolic acid is a potent PPARγ agonist with neuroprotective activity. Br. J. Pharmacol..

[83] Stebulis J.A., Johnson D.R., Rossetti R.G., Burstein S.H., Zurier R.B. (2008). Ajulemic acid, a synthetic cannabinoid acid, induces an antiinflammatory profile of eicosanoids in human synovial cells. Life Sci..

[84] Irrera N., D’ascola A., Pallio G., Bitto A., Mazzon E., Mannino F., Squadrito V., Arcoraci V., Minutoli L., Campo G.M. (2019). β-Caryophyllene Mitigates Collagen Antibody Induced Arthritis (CAIA) in Mice Through a Cross-Talk between CB2 and PPAR-γ Receptors. Biomolecules.

[85] Gonzalez E.G., Selvi E., Balistreri E., Akhmetshina A., Palumbo K., Lorenzini S., Lazzerini P.E., Montilli C., Capecchi P.L., Lucattelli M. (2012). Synthetic cannabinoid ajulemic acid exerts potent antifibrotic effects in experimental models of systemic sclerosis. Ann. Rheum. Dis..

[86] del Rio C., Cantarero I., Palomares B., Cañas M.G., Ruiz J.F., Pavicic C., Martín A.G., Bellido M.L., Castro R.O., Sánchez C.P. (2018). VCE-004.3, a cannabidiol aminoquinone derivative, prevents bleomycin-induced skin fibrosis and inflammation through PPARγ- and CB2 receptor-dependent pathways. Br. J. Pharmacol..

[87] del Río C., Navarrete C., Collado J.A., Bellido M.L., Cañas M.G., Pazos M.R., Ruiz J.F., Pollastro F., Appendino G., Calzado M.A. (2016). The cannabinoid quinol VCE-004.8 alleviates bleomycin-induced scleroderma and exerts potent antifibrotic effects through peroxisome proliferator-activated receptor-γ and CB2 pathways. Sci. Rep..

[88] Martín A.G., Rodríguez M.G., Navarrete C., del Río C., Bellido M.L., Appendino G., Calzado M.A., Muñoz E. (2018). EHP-101, an oral formulation of the cannabidiol aminoquinone VCE-004.8, alleviates bleomycin-induced skin and lung fibrosis. Biochem. Pharmacol..

[89] Navarrete C., Martin A.G., Rodríguez M.G., Mestre L., Feliú A., Guaza C., Calzado M.A., Muñoz E. (2020). Effects of EHP-101 on inflammation and remyelination in murine models of Multiple sclerosis. Neurobiol. Dis..

[90] Navarrete C., Salinas F.C., Palomares B., Mecha M., Jiménez C.J., Mestre L., Feliú A., Bellido M.L., Fiebich B.L., Appendino G. (2018). Hypoxia mimetic activity of VCE-004.8, a cannabidiol quinone derivative: Implications for multiple sclerosis therapy. J. Neuroinflamm..

[91] Lee Y.J., Choi I.S., Park M.H., Lee Y.M., Song J.K., Kim Y.H., Kim K.H., Hwang D.Y., Jeong J.H., Yun Y.P. (2011). 4-O-Methylhonokiol attenuates memory impairment in presenilin 2 mutant mice through reduction of oxidative damage and inactivation of astrocytes and the ERK pathway. Free Radic. Biol. Med..

[92] Cheng Y., Dong Z., Liu S. (2014). β-caryophyllene ameliorates the Alzheimer-like phenotype in APP/PS1 mice through CB2 receptor activation and the PPARγ pathway. Pharmacology.

[93] Ojha S., Javed H., Azimullah S., Haque M.E. (2016). β-Caryophyllene, a phytocannabinoid attenuates oxidative stress, neuroinflammation, glial activation, and salvages dopaminergic neurons in a rat model of Parkinson disease. Mol. Cell. Biochem..

[94] Mansouri S.A., Ojha S., Maamari E.A., Ameri M.A., Nurulain S.M., Bahi A. (2014). The cannabinoid receptor 2 agonist, β-caryophyllene, reduced voluntary alcohol intake and attenuated ethanol-induced place preference and sensitivity in mice. Pharmacol. Biochem. Behav..

[95] Galaj E., Bi G.H., Moore A., Chen K., He Y., Gardner E., Xi Z.X. (2020). Beta-caryophyllene inhibits cocaine self-administration by activation of PPARα and PPARγ: Repurposing a FDA-approved food additive for cocaine use disorder. Neuropsychopharmacology.

[96] Recht L.D., Salmonsen R., Rosetti R., Jang T., Pipia G., Kubiatowski T., Karim P., Ross A.H., Zurier R., Scott Litofsky N. (2001). Antitumor effects of ajulemic acid (CT3), a synthetic non-psychoactive cannabinoid. Biochem. Pharmacol..

[97] Hyun S., Kim M.S., Song Y.S., Bak Y., Ham S.Y., Lee D.H., Hong J., Yoon D.Y. (2015). Peroxisome Proliferator-Activated Receptor-Gamma Agonist 4-O-Methylhonokiol Induces Apoptosis by Triggering the Intrinsic Apoptosis Pathway and Inhibiting the PI3K/Akt Survival Pathway in SiHa Human Cervical Cancer Cells. J. Microbiol. Biotechnol..

[98] Irrera N., D’ascola A., Pallio G., Bitto A., Mannino F., Arcoraci V., Rottura M., Ieni A., Minutoli L., Metro D. (2020). β-caryophyllene inhibits cell proliferation through a direct modulation of CB2 receptors in glioblastoma cells. Cancers.

[99] Hong Y., Zhou Y., Wang Y., Xiao S., Liao D.J., Zhao Q. (2013). PPARγ mediates the effects of WIN55,212-2, an synthetic cannabinoid, on the proliferation and apoptosis of the BEL-7402 hepatocarcinoma cells. Mol. Biol. Rep..

[100] Palomares B., Pino F.R., Navarrete C., Velasco I., Garrido M.A.S., Jimenez C.J., Pavicic C., Vazquez M.J., Appendino G., Bellido M.L. (2018). VCE-004.8, A Multitarget Cannabinoquinone, Attenuates Adipogenesis and Prevents Diet-Induced Obesity. Sci. Rep..

[101] Zhang Z., Chen J., Jiang X., Wang J., Yan X., Zheng Y., Conklin D.J., Kim K.S., Kim K.H., Tan Y. (2014). The magnolia bioactive constituent 4-O-methylhonokiol protects against high-fat diet-induced obesity and systemic insulin resistance in mice. Oxid. Med. Cell. Longev..

[102] Borrelli F., Romano B., Petrosino S., Pagano E., Capasso R., Coppola D., Battista G., Orlando P., Di Marzo V., Izzo A.A. (2015). Palmitoylethanolamide, a naturally occurring lipid, is an orally effective intestinal anti-inflammatory agent. Br. J. Pharmacol..

[103] Hashiesh H.M., Meeran M.F.N., Sharma C., Sadek B., Kaabi J.A., Ojha S.K. (2020). Therapeutic Potential of β-Caryophyllene: A Dietary Cannabinoid in Diabetes and Associated Complications. Nutrients.

[104] Wu K., Xiu Y., Zhou P., Qiu Y., Li Y. (2019). A New Use for an Old Drug: Carmofur Attenuates Lipopolysaccharide (LPS)-Induced Acute Lung Injury via Inhibition of FAAH and NAAA Activities. Front. Pharmacol..

[105] Malamas M.S., Farah S.I., Lamani M., Pelekoudas D.N., Perry N.T., Rajarshi G., Miyabe C.Y., Chandrashekhar H., West J., Pavlopoulos S. (2020). Design and synthesis of cyanamides as potent and selective N-acylethanolamine acid amidase inhibitors. Bioorganic Med. Chem..

[106] Rock E.M., Limebeer C.L., Ward J.M., Cohen A., Grove K., Niphakis M.J., Cravatt B.F., Parker L.A. (2015). Interference with acute nausea and anticipatory nausea in rats by fatty acid amide hydrolase (FAAH) inhibition through a PPARα and CB1 receptor mechanism, respectively: A double dissociation. Psychopharmacology.

[107] Fotio Y., Palese F., Tipan P.G., Ahmed F., Piomelli D. (2020). Inhibition of fatty acid amide hydrolase in the CNS prevents and reverses morphine tolerance in male and female mice. Br. J. Pharmacol..

[108] Ayoub S.M., Smoum R., Farag M., Atwal H., Collins S.A., Rock E.M., Limebeer C.L., Piscitelli F., Iannotti F.A., Lichtman A.H. (2020). Oleoyl alanine (HU595): A stable monomethylated oleoyl glycine interferes with acute naloxone precipitated morphine withdrawal in male rats. Psychopharmacology.

[109] Rock E.M., Ayoub S.M., Limebeer C.L., Gene A., Wills K.L., de Vuono M.V., Smoum R., Di Marzo V., Lichtman A.H., Mechoulam R. (2020). Acute naloxone-precipitated morphine withdrawal elicits nausea-like somatic behaviors in rats in a manner suppressed by N-oleoylglycine. Psychopharmacology.

[110] Donvito G., Piscitelli F., Muldoon P., Jackson A., Vitale R.M., D’Aniello E., Giordano C., Ignatowska-Jankowska B.M., Mustafa M.A., Guida F. (2019). N-Oleoyl-glycine reduces nicotine reward and withdrawal in mice. Neuropharmacology.

[111] Raymundi A.M., da Silva T.R., Zampronio A.R., Guimarães F.S., Bertoglio L.J., Stern C.A.J. (2020). A time-dependent contribution of hippocampal CB1, CB2 and PPARγ receptors to cannabidiol-induced disruption of fear memory consolidation. Br. J. Pharmacol..

[112] Esposito G., Scuderi C., Valenza M., Togna G.I., Latina V., de Filippis D., Cipriano M., Carratù M.R., Iuvone T., Steardo L. (2011). Cannabidiol reduces Aβ-induced neuroinflammation and promotes hippocampal neurogenesis through PPARγ involvement. PLoS ONE.

[113] Fakhfouri G., Ahmadiani A., Rahimian R., Grolla A.A., Moradi F., Haeri A. (2012). WIN55212-2 attenuates amyloid-beta-induced neuroinflammation in rats through activation of cannabinoid receptors and PPAR-γ pathway. Neuropharmacology.

[114] Alonso J.D., Luna J.P., Navarrete C., del Río C., Cantarero I., Palomares B., Aguareles J., Ruiz J.F., Bellido M.L., Pollastro F. (2016). VCE-003.2, a novel cannabigerol derivative, enhances neuronal progenitor cell survival and alleviates symptomatology in murine models of Huntington’s disease. Sci. Rep..

[115] Aguareles J., Luna J.P., Palomares B., Grañeras R.B., Navarrete C., Calvo A.R., Rincón D.G., Taboada E.G., Guzmán M., Muñoz E. (2019). Oral administration of the cannabigerol derivative VCE-003.2 promotes subventricular zone neurogenesis and protects against mutant huntingtin-induced neurodegeneration. Transl. Neurodegener..

[116] García C., Cañas M.G., Burgaz S., Palomares B., Gálvez Y.G., Garo C.P., Campo S., Hernández J.F., Pavicic C., Navarrete C. (2018). Benefits of VCE-003.2, a cannabigerol quinone derivative, against inflammation-driven neuronal deterioration in experimental Parkinson’s disease: Possible involvement of different binding sites at the PPARγ receptor. J. Neuroinflamm..

[117] Burgaz S., García C., Gómez-Cañas M., Muñoz E., Ruiz J.F. (2019). Development of An Oral Treatment with the PPAR-γ-Acting Cannabinoid VCE-003.2 Against the Inflammation-Driven Neuronal Deterioration in Experimental P arkinson’s Disease. Molecules.

[118] Granja A.G., Salinas F.C., Pagani A., Cañas M.G., Negri R., Navarrete C., Mecha M., Mestre L., Fiebich L., Cantarero I. (2012). A Cannabigerol Quinone Alleviates Neuroinflammation in a Chronic Model of Multiple Sclerosis. J. Neuroimmune Pharmacol..

[119] Hind W.H., England T.J., O’Sullivan S.E. (2016). Cannabidiol protects an in vitro model of the blood-brain barrier from oxygen-glucose deprivation via PPARγ and 5-HT1A receptors. Br. J. Pharmacol..

[120] O’Sullivan S.E., Tarling E.J., Bennett A.J., Kendall D.A., Randall M.D. (2005). Novel time-dependent vascular actions of Δ9- tetrahydrocannabinol mediated by peroxisome proliferator-activated receptor gamma. Biochem. Biophys. Res. Commun..

[121] Enayatfard L., Rostami F., Nasoohi S., Oryan S., Ahmadiani A., Dargahi L. (2013). Dual role of PPAR-γ in induction and expression of behavioral sensitization to cannabinoid receptor agonist WIN55,212-2. Neurol. Mol. Med..

[122] Suzuki M.H., Takeda S., Watanabe K., Takiguchi M., Aramaki H. (2019). Δ 9-Tetrahydrocannabinol upregulates fatty acid 2-hydroxylase (FA2H) via PPARα induction: A possible evidence for the cancellation of PPARβ/δ-mediated inhibition of PPARα in MDA-MB-231cells. Arch. Biochem. Biophys..

[123] Payandemehr B., Ebrahimi A., Gholizadeh R., Rahimian R., Varastehmoradi B., Gooshe M., Aghaei H.N., Mousavizadeh K., Dehpour A.R. (2015). Involvement of PPAR receptors in the anticonvulsant effects of a cannabinoid agonist, WIN 55,212-2. Prog. Neuro Psychopharmacol. Biol. Psychiatry.

[124] Couch D.G., Cook H., Ortori C., Barrett D., Lund J.N., O’Sullivan S.E. (2019). Palmitoylethanolamide and Cannabidiol Prevent Inflammation-induced Hyperpermeability of the Human Gut In Vitro and In Vivo—A Randomized, Placebo-controlled, Double-blind Controlled Trial. Inflamm. Bowel Dis..

[125] Rock E.M., Sullivan M.T., Pravato S., Pratt M., Limebeer C.L., Parker L.A. (2020). Effect of combined doses of Δ9-tetrahydrocannabinol and cannabidiol or tetrahydrocannabinolic acid and cannabidiolic acid on acute nausea in male Sprague-Dawley rats. Psychopharmacology.

[126] Bai J., Ge G., Wang Y., Zhang W., Wang Q., Wang W., Guo X., Yu B., Xu Y., Yang H. (2019). A selective CB2 agonist protects against the inflammatory response and joint destruction in collagen-induced arthritis mice. Biomed. Pharmacother..

[127] Fukuda S., Kohsaka H., Takayasu A., Yokoyama W., Miyabe C., Miyabe Y., Harigai M., Miyasaka N., Nanki T. (2014). Cannabinoid receptor 2 as a potential therapeutic target in rheumatoid arthritis. BMC Musculoskelet. Disord..

[128] Lowin T., Schneider M., Pongratz G. (2019). Joints for joints: Cannabinoids in the treatment of rheumatoid arthritis. Curr. Opin. Rheumatol..

[129] Hounoki H., Sugiyama E., Mohamed S.G.K., Shinoda K., Taki H., Abdel-Aziz H.O., Maruyama M., Kobayashi M., Miyahara T. (2008). Activation of peroxisome proliferator-activated receptor γ inhibits TNF-α-mediated osteoclast differentiation in human peripheral monocytes in part via suppression of monocyte chemoattractant protein-1 expression. Bone.

[130] Ji J.D., Cheon H., Jun J.B., Choi S.J., Kim Y.R., Lee Y.H., Kim T.H., Chae I.J., Song G.G., Yoo D.H. (2001). Effects of peroxisome proliferator-activated receptor-γ (PPAR-γ) on the expression of inflammatory cytokines and apoptosis induction in rheumatoid synovial fibroblasts and monocytes. J. Autoimmun..

[131] Liu J., Li H., Burstein S.H., Zurier R.B., Chen J.D. (2003). Activation and binding of peroxisome proliferator-activated receptor γ by synthetic cannabinoid ajulemic acid. Mol. Pharmacol..

[132] Parker J., Atez F., Rossetti R.G., Skulas A., Patel R., Zurier R.B. (2008). Suppression of human macrophage interleukin-6 by a nonpsychoactive cannabinoid acid. Rheumatol. Int..

[133] Zurier R.B., Rossetti R.G., Lane J.H., Goldberg J.M., Hunter S.A., Burstein S.H. (1998). Dimethylheptyl-THC-11 OIC acid: A nonpsychoactive antiinflammatory agent with a cannabinoid template structure. Arthritis Rheum..

[134] Zurier R.B., Rossetti R.G., Burstein S.H., Bidinger B. (2003). Suppression of human monocyte interleukin-1β production by ajulemic acid, a nonpsychoactive cannabinoid. Biochem. Pharmacol..

[135] Burstein S.H., Audette C.A., Doyle S.A., Breuer A., Devane W.A., Colodner S., Mechoulam R. (1992). Synthetic Nonpsychotropic Cannabinoids with Potent Antiinflammatory, Analgesic, and Leukocyte Antiadhesion Activities. J. Med. Chem..

[136] Burstein S. (2005). Ajulemic Acid (IP-751): Synthesis, Proof of Principle, Toxicity Studies, and Clinical Trials. AAPS J..

[137] Karmakar S., Kay J., Gravallese E.M. (2010). Bone Damage in rheumatoid arthritis: Mechanistic insights and approaches to prevention. Rheum. Dis. Clin. N. Am..

[138] George K.L., Saltman L.H., Stein G.S., Lian J.B., Zurier R.B. (2008). Ajulemic acid, a nonpsychoactive cannabinoid acid, suppresses osteoclastogenesis in mononuclear precursor cells and induces apoptosis in mature osteoclast-like cells. J. Cell. Physiol..

[139] Youssef D.A., Fayoumi H.M.E., Mahmoud M.F. (2019). Beta-caryophyllene alleviates diet-induced neurobehavioral changes in rats: The role of CB2 and PPAR-γ receptors. BioMed Pharmacother..

[140] Wu C., Jia Y., Lee J.H., Jun H.J., Lee H.S., Hwang K.Y., Lee S.J. (2014). Trans-Caryophyllene is a natural agonistic ligand for peroxisome proliferator-activated receptor-α. Bioorganic Med. Chem. Lett..

[141] Jiang X., Chen S., Zhang Q., Yi C., He J., Ye X., Liu M., Lu W. (2020). Celastrol is a novel selective agonist of cannabinoid receptor 2 with anti-inflammatory and anti-fibrotic activity in a mouse model of systemic sclerosis. Phytomedicine.

[142] Servettaz A., Kavian N., Nicco C., Deveaux V., Chéreau C., Wang A., Zimmer A., Lotersztajn S., Weill B., Batteux F. (2010). Targeting the cannabinoid pathway limits the development of fibrosis and autoimmunity in a mouse model of systemic sclerosis. Am. J. Pathol..

[143] Gonzalez E.G., Selvi E., Balistreri E., Lorenzini S., Maggio R., Natale M.R., Capecchi P.L., Lazzerini P.E., Bardelli M., Laghi-Pasini F. (2009). Cannabinoids inhibit fibrogenesis in diffuse systemic sclerosis fibroblasts. Rheumatology.

[144] Dantas A.T., Pereira M.C., de Melo Rego M.J.B., da Rocha L.F., Pitta I.D.R., Marques C.D.L., Duarte A.L.B.P., Pitta M.G.D.R. (2015). The Role of PPAR Gamma in Systemic Sclerosis. PPAR Res..

[145] Lucattelli M., Fineschi S., Selvi E., Gonzalez E.G., Bartalesi B., de Cunto G., Lorenzini S., Galeazzi M., Lungarella G. (2016). Ajulemic acid exerts potent anti-fibrotic effect during the fibrogenic phase of bleomycin lung. Respir. Res..

[146] Spiera R., Hummers L., Chung L., Frech T.M., Domsic R., Hsu V., Furst D.E., Gordon J., Mayes M., Simms R. (2020). Safety and Efficacy of Lenabasum in a Phase II, Randomized, Placebo-Controlled Trial in Adults with Systemic Sclerosis. Arthritis Rheumatol..

[147] To Evaluate the Safety and Tolerability, Pharmacokinetics, Food-effect and Pharmacodynamics of EHP-101 in Healthy Volunteers. https://www.clinicaltrials.gov/ct2/show/NCT03745001?term=EHP-101&draw=2&rank=2.

[148] Alberti T.B., Barbosa W.L.R., Vieira J.L.F., Raposo N.R.B., Dutra R.C. (2017). (−)-β-caryophyllene, a CB2 receptor-selective phytocannabinoid, suppresses motor paralysis and neuroinflammation in a murine model of multiple sclerosis. Int. J. Mol. Sci..

[149] Fontes L.B.A., Dias D., Dos S., Aarestrup B.J.V., Aarestrup F.M., da Silva Filho A.A., do Corrêa J.O.A. (2017). β-Caryophyllene ameliorates the development of experimental autoimmune encephalomyelitis in C57BL/6 mice. BioMed Pharmacother..

[150] Askari V.R., Nick R.S. (2019). Promising neuroprotective effects of β-caryophyllene against LPS-induced oligodendrocyte toxicity: A mechanistic study. Biochem. Pharmacol..

[151] Lee Y.K., Choi I.S., Ban J.O., Lee H.J., Lee U.S., Han S.B., Jung J.K., Kim Y.H., Kim K.H., Oh K.W. (2011). 4-O-methylhonokiol attenuated β-amyloid-induced memory impairment through reduction of oxidative damages via inactivation of p38 MAP kinase. J. Nutr. Biochem..

[152] Choi I.S., Lee Y.J., Choi D.Y., Lee Y.K., Lee Y.H., Kim K.H., Kim Y.H., Jeon Y.H., Kim E.H., Han S.B. (2011). 4-O-methylhonokiol attenuated memory impairment through modulation of oxidative damage of enzymes involving amyloid-β generation and accumulation in a mouse model of alzheimer’s disease. J. Alzheimer Dis..

[153] Lee Y.J., Choi D.Y., Lee Y.K., Lee Y.M., Han S.B., Kim Y.H., Kim K.H., Nam S.Y., Lee B.J., Kang J.K. (2012). 4-O-methylhonokiol prevents memory impairment in the tg2576 transgenic mice model of alzheimer’s disease via regulation of β-secretase activity. J. Alzheimer Dis..

[154] Jung Y.-Y., Lee Y.-J., Choi D.-Y., Hong J.T. (2014). Amelioration of Cognitive Dysfunction in APP/PS1 Double Transgenic Mice by Long-Term Treatment of 4-O-Methylhonokiol. Biomol. Ther..

[155] Lee Y.J., Choi D.Y., Choi I.S., Kim K.H., Kim Y.H., Kim H.M., Lee K., Cho W.G., Jung J.K., Han S.B. (2012). Inhibitory effect of 4-O-methylhonokiol on lipopolysaccharide-induced neuroinflammation, amyloidogenesis and memory impairment via inhibition of nuclear factor-kappaB in vitro and in vivo models. J. Neuroinflamm..

[156] Askari V.R., Nick R.S. (2019). The protective effects of β-caryophyllene on LPS-induced primary microglia M_1_/M_2_ imbalance: A mechanistic evaluation. Life Sci..

[157] Hu Y., Zeng Z., Wang B., Guo S. (2017). Trans-caryophyllene inhibits amyloid β (Aβ) oligomer-induced neuroinflammation in BV-2 microglial cells. Int. Immunopharmacol..

[158] Schuehly W., Paredes J.M.V., Kleyer J., Huefner A., Goffer S.A., Raduner S., Altmann K.-H., Gertsch J. (2011). Mechanisms of osteoclastogenesis inhibition by a novel class of biphenyl-type cannabinoid CB (2) receptor inverse agonists. Chem. Biol..

[159] Han Y., Liu J., Ahn S., An S., Ko H., Shin J.C., Jin S.H., Ki M.W., Lee S.H., Lee K.H. (2020). Diallyl biphenyl-type neolignans have a pharmacophore of pparα/γ dual modulators. Biomol. Ther..

[160] Javed H., Azimullah S., Haque M.E., Ojha S.K. (2016). Cannabinoid type 2 (CB2) receptors activation protects against oxidative stress and neuroinflammation associated dopaminergic neurodegeneration in rotenone model of parkinson’s disease. Front. Neurosci..

[161] Paredes J.M.V., Castañeda R.E.G., Gertsch J., Huerta V.C., Roa R.I.L., Valls E.V., Zarate C.B., Espuny A.C., Soto M.E.F. (2017). Neuroprotective Effects of β-caryophyllene against dopaminergic neuron injury in a murine model of parkinson’s disease induced by MPTP. Pharmaceuticals.

[162] Wang G., Ma W., Du J. (2018). β-Caryophyllene (BCP) ameliorates MPP+ induced cytotoxicity. BioMed Pharmacother..

[163] Smith J.P., Book S.W. (2008). Anxiety and substance use disorders: A review. Psychiatr. Times.

[164] Bahi A., Mansouri S.A., Memari E.A., Ameri M.A., Nurulain S.M., Ojha S. (2014). β-Caryophyllene, a CB2 receptor agonist produces multiple behavioral changes relevant to anxiety and depression in mice. Physiol. Behav..

[165] Schwarz R., Ramer R., Hinz B. (2018). Targeting the endocannabinoid system as a potential anticancer approach. Drug Metab. Rev..

[166] Gómez E.P., Andradas C., Benito S.B., Caffarel M.M., Taboada E.G., Morales M.V., Moreno E., Hamann S., Villar E.M., Flores J.M. (2015). Role of cannabinoid receptor CB2 in HER2 pro-oncogenic signaling in breast cancer. J. Natl. Cancer Inst..

[167] McKallip R.J., Lombard C., Fisher M., Martin B.R., Ryu S., Grant S., Nagarkatti P.S., Nagarkatti M. (2002). Targeting CB2 cannabinoid receptors as a novel therapy to treat malignant lymphoblastic disease. Blood.

[168] Sánchez C., del Pulgar T.G., Rueda D., Velasco G., Roperh I.G., Guzmán M., de Ceballos M.L., Corbacho C., Ramón y Cajal S., Huffman J.W. (2001). Inhibition of glioma growth in vivo by selective activation of the CB2 cannabinoid receptor. Cancer Res..

[169] Burstein S.H. (2018). Ajulemic acid: Potential treatment for chronic inflammation. Pharmacol. Res. Perspect..

[170] Garg N., Smith T.W. (2015). An update on immunopathogenesis, diagnosis, and treatment of multiple sclerosis. Brain Behav..

[171] Lee N., Oh J., Ban J., Shim J., Lee H., Jung J., Ahn B., Yoon D., Han S., Ham Y. (2013). 4-O-methylhonokiol, a PPARγ agonist, inhibits prostate tumour growth: p21-mediated suppression of NF-κB activity. Br. J. Pharmacol..

[172] Cho J.H., Lee R.H., Jeon Y.J., Shin J.C., Park S.M., Choi N.J., Seo K.S., Yoon G., Cho S.S., Kim K.H. (2015). Role of transcription factor Sp1 in the 4-O-methylhonokiol-mediated apoptotic effect on oral squamous cancer cells and xenograft. Int. J. Biochem. Cell Biol..

[173] Xiao S., Chen F., Gao C. (2017). Antitumor activity of 4-O-Methylhonokiol in human oral cancer cells is mediated via ROS generation, disruption of mitochondrial potential, cell cycle arrest and modulation of Bcl-2/Bax proteins. JBUON.

[174] Oh J.H., Ban J.O., Cho M.C., Jo M., Jung J.K., Ahn B., Yoon D.Y., Han S.B., Hong J.T. (2012). 4-O-methylhonokiol inhibits colon tumor growth via p21-mediated suppression of NF-κB activity. J. Nutr. Biochem..

[175] Hall J.A., Rusten M., Abughazaleh R.D., Wuertz B., Souksavong V., Escher P., Ondrey F. (2020). Effects of PPAR-γ agonists on oral cancer cell lines: Potential horizons for chemopreventives and adjunctive therapies. Head Neck.

[176] Ban J.O., Kwak D.H., Oh J.H., Park E.J., Cho M.C., Song H.S., Song M.J., Han S.B., Moon D.C., Kang K.W. (2010). Suppression of NF-κB and GSK-3β is involved in colon cancer cell growth inhibition by the PPAR agonist troglitazone. Chem. Biol. Interact..

[177] Kumawat V.S., Kaur G. (2020). Insulinotropic and antidiabetic effects of β-caryophyllene with l-arginine in type 2 diabetic rats. J. Food Biochem..

[178] Suijun W., Zhen Y., Ying G., Yanfang W. (2014). A role for trans-caryophyllene in the moderation of insulin secretion. Biochem. Biophys. Res. Commun..

[179] Youssef D.A., Fayoumi H.M.E., Mahmoud M.F. (2019). Beta-caryophyllene protects against diet-induced dyslipidemia and vascular inflammation in rats: Involvement of CB2 and PPAR-γ receptors. Chem. Biol. Interact..

[180] Kaur G., Tharappel L.J.P., Kumawat V. (2018). Evaluation of safety and in vitro mechanisms of anti-diabetic activity of β-caryophyllene and L-arginine. J. Biol. Sci..

[181] Varga Z.V., Matyas C., Erdelyi K., Cinar R., Nieri D., Chicca A., Nemeth B.T., Paloczi J., Lajtos T., Corey L. (2018). β-Caryophyllene protects against alcoholic steatohepatitis by attenuating inflammation and metabolic dysregulation in mice. Br. J. Pharmacol..

[182] Scandiffio R., Geddo F., Cottone E., Querio G., Antoniotti S., Gallo M.P., Maffei M.E., Bovolin P. (2020). Protective Effects of (E)-β-Caryophyllene (BCP) in Chronic Inflammation. Nutrients.

[183] Rossi F., Punzo F., Umano G.R., Argenziano M., del Giudice E.M. (2018). Role of cannabinoids in obesity. Int. J. Mol. Sci..

[184] Ma T., Zheng Z., Guo H., Lian X., Rane M.J., Cai L., Kim K.S., Kim K.T., Zhang Z., Bi L. (2019). 4-O-methylhonokiol ameliorates type 2 diabetes-induced nephropathy in mice likely by activation of AMPK-mediated fatty acid oxidation and Nrf2-mediated anti-oxidative stress. Toxicol. Appl. Pharmacol..

[185] Zheng Z., Ma T., Guo H., Kim K.S., Kim K.T., Bi L., Zhang Z., Cai L. (2019). 4-O-methylhonokiol protects against diabetic cardiomyopathy in type 2 diabetic mice by activation of AMPK-mediated cardiac lipid metabolism improvement. J. Cell. Mol. Med..

[186] Patsenker E., Chicca A., Petrucci V., Moghadamrad S., de Gottardi A., Hampe J., Gertsch J., Semmo N., Stickel F. (2017). 4-O′-methylhonokiol protects from alcohol/carbon tetrachloride-induced liver injury in mice. J. Mol. Med..

[187] Deutsch D.G., Chin S.A. (1993). Enzymatic synthesis and degradation of anandamide, a cannabinoid receptor agonist. Biochem. Pharmacol..

[188] Cravatt B.F., Demarest K., Patricelli M.P., Bracey M.H., Giang D.K., Martin B.R., Lichtman A.H. (2001). Supersensitivity to anandamide and enhanced endogenous cannabinoid signaling in mice lacking fatty acid amide hydrolase. Proc. Natl. Acad. Sci. USA.

[189] di Marzo V., Melck D., Bisogno T., de Petrocellis L. (1998). Endocannabinoids: Endogenous cannabinoid receptor ligands with neuromodulatory action. Trends Neurosci..

[190] Brunetti L., Loiodice F., Piemontese L., Tortorella P., Laghezza A. (2019). New Approaches to Cancer Therapy: Combining Fatty Acid Amide Hydrolase (FAAH) Inhibition with Peroxisome Proliferator-Activated Receptors (PPARs) Activation. J. Med. Chem..

[191] Caprioglio D., Mattoteia D., Pollastro F., Negri R., Lopatriello A., Chianese G., Minassi A., Collado J.A., Munoz E., Taglialatela-Scafati O. (2020). The Oxidation of Phytocannabinoids to Cannabinoquinoids. J. Nat. Prod..

[192] Scafati O.T., Pagani A., Scala F., de Petrocellis L., di Marzo V., Grassi G., Appendino G. (2010). Cannabimovone, a cannabinoid with a rearranged terpenoid skeleton from hemp. Eur. J. Org. Chem..

[193] Iannotti F.A., de Maio F., Panza E., Appendino G., Scafati O.T., de Petrocellis L., Amodeo P., Vitale R.M. (2020). Identification and Characterization of Cannabimovone, a Cannabinoid from Cannabis sativa, as a Novel PPARγ Agonist via a Combined Computational and Functional Study. Molecules.

[194] Sanz G.M. (2016). Can You Pass the Acid Test? Critical Review and Novel Therapeutic Perspectives of Δ9-Tetrahydrocannabinolic Acid A. Cannabis Cannabinoid Res..

[195] Raman P., Kaplan B.L.F., Thompson J.T., Heuvel J.P.V., Kaminski N.E. (2011). Metabolite of 2-Arachidonyl Glycerol, Activates Peroxisome Proliferator Activated Receptor gamma. Mol. Pharmacol..

[196] Fakhrudin N., Ladurner A., Atanasov A.G., Heiss E.H., Baumgartner L., Markt P., Schuster D., Ellmerer E.P., Wolber G., Rollinger J.M. (2010). Computer-aided discovery, validation, and mechanistic characterization of novel neolignan activators of peroxisome proliferator-activated receptor γ. Mol. Pharmacol..

[197] Zhang J., Chen Z., Huang X., Shi W., Zhang R., Chen M., Huang H., Wu L. (2019). Insights on the Multifunctional Activities of Magnolol. BioMed Res. Int..

[198] Lin M.H., Chen M.C., Chen T.H., Chang H.Y., Chou T.C. (2015). Magnolol ameliorates lipopolysaccharide-induced acute lung injury in rats through PPAR-γ-dependent inhibition of NF-kB activation. Int. Immunopharmacol..

[199] Shen P., Zhang Z., He Y., Gu C., Zhu K., Li S., Li Y., Lu X., Liu J., Zhang N. (2018). Magnolol treatment attenuates dextran sulphate sodium-induced murine experimental colitis by regulating inflammation and mucosal damage. Life Sci..

[200] Xie Z., Zhao J., Wang H., Jiang Y., Yang Q., Fu Y., Zeng H., Hölscher C., Xu J., Zhang Z. (2020). Magnolol alleviates Alzheimer’s disease-like pathology in transgenic C. elegans by promoting microglia phagocytosis and the degradation of beta-amyloid through activation of PPAR-γ. BioMed Pharmacother..

[201] Shih C.Y., Chou T.C. (2012). The antiplatelet activity of magnolol is mediated by PPAR-β/γ. Biochem. Pharmacol..

[202] Liang X., Xing W., He J., Fu F., Zhang W., Su F., Liu F., Ji L., Gao F., Su H. (2015). Magnolol administration in normotensive young spontaneously hypertensive rats postpones the development of hypertension: Role of increased PPAR gamma, reduced TRB3 and resultant alleviative vascular insulin resistance. PLoS ONE.

[203] Choi S.S., Cha B.Y., Lee Y.S., Yonezawa T., Teruya T., Nagai K., Woo J.T. (2009). Magnolol enhances adipocyte differentiation and glucose uptake in 3T3-L1 cells. Life Sci..

[204] Rempel V., Fuchs A., Hinz S., Karcz T., Lehr M., Koetter U., Müller C.E. (2013). Magnolia extract, magnolol, and metabolites: Activation of cannabinoid CB2 receptors and blockade of the related GPR55. ACS Med. Chem. Lett..

[205] Fuchs A., Rempel V., Müller C.E. (2013). The natural product magnolol as a lead structure for the development of potent cannabinoid receptor agonists. PLoS ONE.

[206] D’Aniello E., Fellous T., Iannotti F.A., Gentile A., Allarà M., Balestrieri F., Gray R., Amodeo P., Vitale R.M., Di Marzo V. (2019). Identification and characterization of phytocannabinoids as novel dual PPARα/γ agonists by a computational and in vitro experimental approach. Biochim. Biophys. Acta Gen. Subj..

[207] Priestley R.S., Nickolls S.A., Alexander S.P.H., Kendall D.A. (2015). A potential role for cannabinoid receptors in the therapeutic action of fenofibrate. FASEB J..

[208] McGuinness D., Malikzay A., Visconti R., Lin K., Bayne M., Monsma F., Lunn C.A. (2009). Characterizing cannabinoid CB2 receptor ligands using DiscoveRx PathHunter^TM^ β-arrestin assay. J. Biomol. Screen..

[209] Verme J.L., Fu J., Astarita G., Rana G.L., Russo R., Calignano A., Piomelli D. (2005). The nuclear receptor peroxisome proliferator-activated receptor-α mediates the anti-inflammatory actions of palmitoylethanolamide. Mol. Pharmacol..

[210] Ambrosio A.L.B.B., Dias S.M.G.G., Polikarpov I., Zurier R.B., Burstein S.H., Garratt R.C. (2007). Ajulemic acid, a synthetic nonpsychoactive cannabinoid acid, bound to the ligand binding domain of the human peroxisome proliferator-activated receptor. J. Biol. Chem..

[211] Tepper M.A., Zurier R.B., Burstein S.H. (2014). Ultrapure ajulemic acid has improved CB2 selectivity with reduced CB1 activity. Bioorg. Med. Chem..

[212] Martín A.G., Rodríguez M.G., Navarrete C., Caprioglio D., Palomares B., de Mesa J., Rollland A., Appendino G., Muñoz E. (2019). Cannabinoid derivatives acting as dual PPARγ/CB2 agonists as therapeutic agents for systemic sclerosis. Biochem. Pharmacol..

[213] Jhaveri M.D., Richardson D., Robinson I., Garle M.J., Patel A., Sun Y., Sagar D.R., Bennett A.J., Alexander S.P.H., Kendall D.A. (2008). Inhibition of fatty acid amide hydrolase and cyclooxygenase-2 increases levels of endocannabinoid related molecules and produces analgesia via peroxisome proliferator-activated receptor-alpha in a model of inflammatory pain. Neuropharmacology.

[214] Morales P., Hurst D.P., Reggio P.H., Kinghorn A.D., Gibbons S. (2017). Molecular Targets of the Phytocannabinoids: A Complex Picture. Phytocannabinoids: Unraveling the Complex Chemistry and Pharmacology of Cannabis Sativa.

[215] Trial to Evaluate Efficacy and Safety of Lenabasum in Dermatomyositis. https://clinicaltrials.gov/ct2/show/NCT03813160?term=lenabasum&draw=2&rank=2.

[216] Kroker A.J., Bruning J.B. (2015). Review of the structural and dynamic mechanisms of PPAR γ partial agonism. PPAR Res..

[217] Itoh T., Fairall L., Amin K., Inaba Y., Szanto A., Balint B.L., Nagy L., Yamamoto K., Schwabe J.W.R. (2008). Structural basis for the activation of PPARγ by oxidized fatty acids. Nat. Struct. Mol. Biol..

[218] Chandra V., Huang P., Hamuro Y., Raghuram S., Wang Y., Burris T.P., Rastinejad F. (2008). Structure of the intact PPAR-γ–RXR-α nuclear receptor complex on DNA. Nature.

[219] Cronet P., Petersen J.F.W., Folmer R., Blomberg N., Sjöblom K., Karlsson U., Lindstedt E.L., Bamberg K. (2001). Structure of the PPARα and -γ ligand binding domain in complex with AZ 242; ligand selectivity and agonist activation in the PPAR family. Structure.

[220] Mileni M., Kamtekar S., Wood D.C., Benson T.E., Cravatt B.F., Stevens R.C. (2010). Crystal structure of fatty acid amide hydrolase bound to the carbamate inhibitor URB597: Discovery of a deacylating water molecule and insight into enzyme inactivation. J. Mol. Biol..

[221] van Gastel J., Hendrickx J.O., Leysen H., Otte P.S., Luttrell L.M., Martin B., Maudsley S. (2018). β-Arrestin based receptor signaling paradigms: Potential therapeutic targets for complex age-related disorders. Front. Pharmacol..

[222] Miljuš T., Heydenreich F.M., Gazzi T., Kimbara A., Nettekoven M., Zirwes E., Osterwald A., Rufer A.C., Ullmer C., Guba W. (2020). Diverse chemotypes drive biased signaling by cannabinoid. bioRxiv.

[223] Zoubi R.A., Morales P., Reggio P.H. (2019). Structural Insights into CB1 Receptor Biased Signaling. Int. J. Mol. Sci..

[224] Morales P., Goya P., Jagerovic N. (2018). Emerging strategies targeting CB2 cannabinoid receptor: Biased agonism and allosterism. Biochem. Pharmacol..

[225] Kang J., Shi Y., Xiang B., Qu B., Su W., Zhu M., Zhang M., Bao G., Wang F., Zhang X. (2005). A nuclear function of β-arrestin1 in GPCR signaling: Regulation of histone acetylation and gene transcription. Cell.

[226] Hoeppner C.Z., Cheng N., Ye R.D. (2012). Identification of a nuclear localization sequence in β-arrestin-1 and its functional implications. J. Biol. Chem..

[227] Wang C., Zeng X., Zhou Z., Zhao J., Pei G. (2016). β-arrestin-1 contributes to brown fat function and directly interacts with PPARα and PPARγ. Sci. Rep..

[228] Zhuang L.N., Hu W.X., Xin S.M., Zhao J., Pei G. (2011). β-arrestin-1 protein represses adipogenesis and inflammatory responses through its interaction with peroxisome proliferator-activated receptor-γ (PPARγ). J. Biol. Chem..

[229] Gorgulla C., Padmanabha Das K.M., Leigh K.E., Cespugli M., Fischer P.D., Wang Z.F., Tesseyre G., Pandita S., Shnapir A., Calderaio A. (2020). A multi-pronged approach targeting SARS-CoV-2 proteins using ultra-large virtual screening. ChemRxiv.

